# Functional mapping of N-terminal residues in the yeast proteome uncovers novel determinants for mitochondrial protein import

**DOI:** 10.1371/journal.pgen.1010848

**Published:** 2023-08-16

**Authors:** Salomé Nashed, Houssam El Barbry, Médine Benchouaia, Angélie Dijoux-Maréchal, Thierry Delaveau, Nadia Ruiz-Gutierrez, Lucie Gaulier, Déborah Tribouillard-Tanvier, Guillaume Chevreux, Stéphane Le Crom, Benoit Palancade, Frédéric Devaux, Elodie Laine, Mathilde Garcia

**Affiliations:** 1 Sorbonne Université, CNRS, Institut de Biologie Paris-Seine, UMR 7238, Laboratoire de Biologie Computationnelle et Quantitative, Paris, France; 2 Université de Bordeaux, CNRS, IBGC, UMR5095, Bordeaux, France; 3 Université Paris Cité, CNRS, Institut Jacques Monod, Paris, France; Pacific Northwest Research Institute, UNITED STATES

## Abstract

N-terminal ends of polypeptides are critical for the selective co-translational recruitment of N-terminal modification enzymes. However, it is unknown whether specific N-terminal signatures differentially regulate protein fate according to their cellular functions. In this work, we developed an *in-silico* approach to detect functional preferences in cellular N-terminomes, and identified in *S*. *cerevisiae* more than 200 Gene Ontology terms with specific N-terminal signatures. In particular, we discovered that Mitochondrial Targeting Sequences (MTS) show a strong and specific over-representation at position 2 of hydrophobic residues known to define potential substrates of the N-terminal acetyltransferase NatC. We validated mitochondrial precursors as co-translational targets of NatC by selective purification of translating ribosomes, and found that their N-terminal signature is conserved in *Saccharomycotina* yeasts. Finally, systematic mutagenesis of the position 2 in a prototypal yeast mitochondrial protein confirmed its critical role in mitochondrial protein import. Our work highlights the hydrophobicity of MTS N-terminal residues and their targeting by NatC as important features for the definition of the mitochondrial proteome, providing a molecular explanation for mitochondrial defects observed in yeast or human NatC-depleted cells. Functional mapping of N-terminal residues thus has the potential to support the discovery of novel mechanisms of protein regulation or targeting.

## Author summary

Mitochondria play a central role in eukaryotic cells and defects in their biogenesis are implicated in many serious human diseases. Most mitochondrial proteins are encoded in the nucleus, translated in the cytoplasm and directed to their final destination by N-terminal extensions, called mitochondrial targeting sequences (MTS). A good efficiency of this import mechanism is critical for mitochondrial function and for the success of gene therapies involving the replacement in the nucleus of a defective mitochondrial gene by a functional non-mutated version. Through a systematic analysis of amino acid preferences in yeasts, we observed a strong and specific overrepresentation of hydrophobic residues at position 2 of MTSs that are known to avoid cleavage of the hydrophobic initiator methionine and to define potential substrates of the N-terminal acetyltransferase NatC. Therefore, most MTSs have two consecutive hydrophobic residues at position 1 and 2, a feature that is conserved throughout evolution. Using CRISPR/Cas9 technology, we showed that mutation of mitochondrial proteins at position 2 reduced their import efficiency. We thus demonstrated that the residue at position 2 of MTS is an important determinant of mitochondrial import under strong selective pressure. These findings may have valuable implications for improving the therapy of human mitochondrial diseases.

## Introduction

As soon as they emerge from the ribosomal tunnel, the protein nascent chains recruit factors that will play key roles in their life cycle [1–5]. Such recruitment is dependent on the nature of the protein N-terminal amino acid residues. In particular, the specific recognition and co-translational action of several N-terminal modification enzymes is determined by the type of the amino acid residue directly following the initiator methionine, namely the residue at position 2.

For instance, methionine aminopeptidases (MetAPs) will remove the initiator methionine (iMet) in a large fraction of the nascent chains displaying an amino acid with a small radius of gyration (Ala, Cys, Gly, Pro, Ser, Thr, or Val) at position 2 [6]. In addition, N-terminal acetyltransferases (NATs) catalyze the irreversible covalent attachment of an acetyl group (CH_3_CO) to the free α-amino group (NH_3_^+^) at the protein N-terminus. Three major NATs, namely NatA, NatB and NatC, that acetylate nascent chains in a co-translational manner, are conserved from yeast to human [7]. The actual impact of the inactivation of NATs on the N-terminal acetylation of subsets of cellular proteins was measured by various proteomics techniques, eventually providing experimental data on the N-terminal acetylation status for up to 10% of the cellular proteome in yeast and in human [8–12]. All these studies led to the conclusion that NatA can acetylate the N-termini beginning with Ala, Cys, Gly, Ser, Thr, or Val after removal of iMet, whereas the other two can acetylate the uncleaved iMet if it is followed by a second specific residue, namely Asn, Asp, Gln, or Glu for NatB, and Ile, Leu, Phe, or Trp for NatC. Based on these substrate specificities together with the observed frequencies of the 20 amino acids at position 2 in proteomes and the partial N-terminal acetylome data, it was estimated that 80–90% of human and 50–70% of yeast proteins could potentially be acetylated by one of these three enzymes [7,13,14]. Additionally, Methyl-, myristoyl-, and palmitoyltransferases can also selectively modify, during or after translation, the N-terminus of proteins after iMet cleavage [5]. Finally, after protein cleavage by diverse peptidases including MetAPs, arginine can be post-translationally added by Arginyl-tRNA transferases at the N-terminal end of proteins exposing specific N-terminal residue such as Cys, Asp and Glu [15].

The biological importance of these N-terminal modifications is underlined by the strong defects observed when the corresponding enzymes are inactivated. Complete inactivation of MetAPs activity is lethal in *Escherichia coli*, *Salmonella typhimurium*, and *Saccharomyces cerevisiae*, and the identification of human MetAP1 and MetAP2 as targets for putative anticancer drugs further confirmed the importance of this enzyme family [16,17]. In addition, N-terminal acetylation has been implicated in several diseases, including cancers, developmental disorders, as well as Parkinson’s disease in humans [7], and also in defects in photosynthesis and growth in plant [18]. At the molecular level, the importance of the nature of the second residue and the associated N-terminal modifications have been implicated in all major steps of proteins life cycle including folding and aggregation, protein interactions and complex formation, protein subcellular targeting, and protein turnover through proteasomal degradation pathways (reviewed in 7). In particular, during the last decades, the exploration of the N-end rule pathway, linking the nature of the N-terminus to protein *in vivo* stability has emphasized the importance of protein N-end signatures and demonstrated that iMet cleavage and N-terminal acetylation are important players in the early control of proteins life cycle [15,19–22]. Finally, preferences for the use of amino acids at position 2, different from those observed globally in the proteome, have been described in various species, suggesting that selection pressures act on this particular protein position. In addition, these biases are taxon-specific, indicating progressive changes during evolution [23].

This extensive knowledge on the general importance of protein N-terminal signatures sharply contrasts with the lack of data describing their potential implication in specific functional pathways. To address this issue, we have developed an *in-silico* approach at the proteome scale allowing for the systematic detection of functional biases in the use of the 20 amino acids at position 2 of proteins. The rationale of our approach is that, given the importance of the residue at position 2 in the life cycle of proteins, evolutionary constraints could have led to the selection of specific amino acids at this position in groups of proteins from the same cellular compartment or involved in the same biological pathway. As a proof of concept, we analyzed the *S*. *cerevisiae* proteome to statistically assess all significant and specific overrepresentations of one or more amino acids at position 2 in protein subsets sharing the same Gene Ontology (GO) annotations. Using this approach, we were able to identify various groups of proteins with common GO annotations and characterized by particular amino acid preferences at position 2. We hypothesized that these newly identified N-terminal signatures are likely associated with important functional roles. We further characterized, with a combination of *in-silico* analyses and experimental assays, the function of the strong position-specific bias detected at position 2 for mitochondrial proteins.

Most mitochondrial proteins are translated by cytoplasmic ribosomes and addressed to the mitochondrial compartment via a cleavable presequence, the so-called Mitochondrial Targeting Sequence (MTS). We found that, throughout the *Saccharomycotina* yeast lineage, MTS-bearing mitochondrial precursors present, just after the iMet, predominantly hydrophobic amino acids known to define potential NatC substrates (i.e., Leu, Phe, Ile, and Trp). Affinity-selective purification of the translating ribosomes then confirmed their co-translational recognition by this N-terminal acetyltransferase. Finally, we demonstrated the functional significance of the bias detected at position 2 of MTSs by site-directed mutagenesis of this position in a dominant negative allele of the essential mitochondrial protein Hsp60p. Only the amino acids found to be overrepresented at position 2 of MTSs in our *in-silico* analyses allowed the efficient mitochondrial import of the toxic Hsp60p, as revealed by the associated loss of cell viability.

Our work has revealed an unknown and critical feature of MTSs. Defects in mitochondrial biogenesis are implicated in a number of serious pathologies, including neuropathies, cardiovascular disorders, myopathies, neurodegenerative diseases and cancers [24]. In a long-term perspective, understanding the role of this signature for mitochondrial protein import is therefore crucial for human health. For example, this knowledge could be very useful for the design of mitochondrial gene therapies based on the targeting of specific proteins to the mitochondrial compartment [25]. Beyond the results obtained for the proteins of the mitochondrial compartment, we provide the community with the first functional mapping of N-terminal residues, whose future exploration will undoubtedly contribute to the discovery of novel mechanisms of protein regulation or targeting.

## Results

### Detection of functional biases in amino acid usage at position 2 in *S*. *cerevisiae* proteome

**Fig 1:**
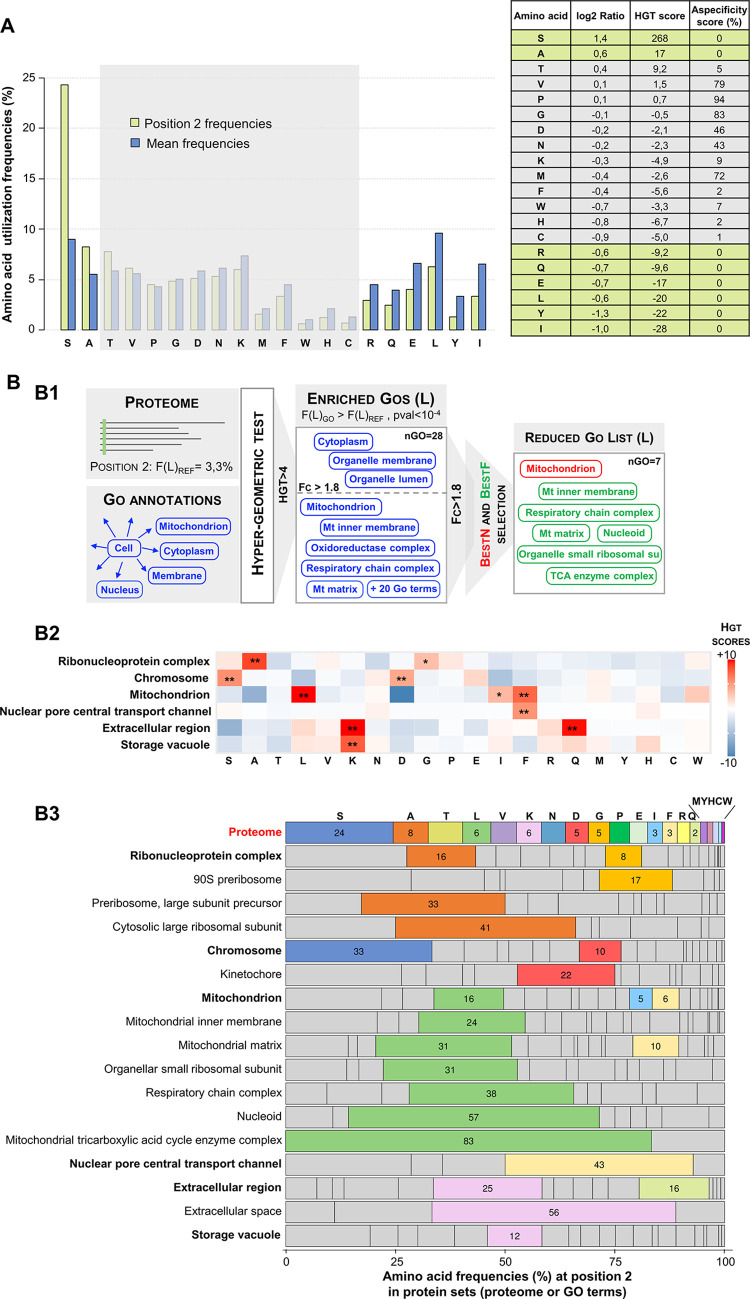
Specific amino acids are over-represented at position 2 in *S*. *cerevisiae* proteome. (A) Serine is the most represented protein at position 2 in *S*. *cerevisiae* proteome. Frequencies (Barplot, left) of the 20 amino acids at position 2 (green bars) are compared to their average usage at any position in *S*. *cerevisiae* proteome (blue bars). Table (right) recapitulates quantitative values calculated to identify significant amino acid usage biases at position 2: position2/mean log2Ratio, HGT score obtained from hypergeometric tests, and aspecificity scores (see Methods). Amino acids for which the absolute value of the HGT score is greater than 3 (p-value<10^−3^) and the aspecificity score is lower than 5% are highlighted. Such values indicate a significant and specific bias of usage at position 2 revealing a possible selection pressure. (B) Several protein groups defined by their annotation in the Gene Ontology database have a specific N-terminal signature. (B1) GO term selection procedure illustrated on the particular case of N-terminal preferences for leucine. Twenty-eight GO terms describing components were extracted on the basis of their significant overrepresentation of leucine at position 2 assessed by hypergeometric test (HGT score > 4). After several filters, 7 of them were finally retained, including a single GO BestN term and 6 GO BestF terms (see more details in S1 Supplementary Methods and S2 Fig). (B2) Heatmap of HGT scores for the 6 BestN GO terms that best describe the GO terms selected in the "components" classification when applying this strategy to the 20 amino acids. Stars indicate significant HGT scores (*HGT>3, **HGT>4). (B3) Bar graph showing the percentages of utilization of the 20 amino acids at position 2 in all selected BestN and BestF GO terms in the "components" classification. Significant amino acid overrepresentation (HGT score > 3) are highlighted. Amino acids are sorted in decreasing order of use at position 2 in the proteome.

The amino acid usage at position 2 of *S*. *cerevisiae* proteins (Fig 1A) is clearly different from the average amino acid distribution in its proteome. Strikingly, we observed a serine at this position in nearly 25% of the yeast proteins. This overrepresentation is both highly significant (HGT score equal to 268 corresponding to a p-value after hypergeometric test equal to 10^−268^, see Methods for a definition of HGT score) and specific to position 2 (aspecificity score of 0%, see Methods). By contrast, several amino acids are underrepresented (HGT score <-3 and aspecificity score <5%), including the hydrophobic residues leucine, isoleucine, and tyrosine as well as charged or polar residues such as arginine, glutamate and glutamine. Such asymmetric distribution, with the over-representation of serine, is conserved in budding yeasts (S1A Fig), as indicated by proteome analysis of 17 yeasts spanning nearly 400 million years of evolution of the *Saccharomycotina* lineage [26,27].

We further developed a computational approach to explore the functional relevance of amino acid usage biases at the proteome scale and assess their statistical significance (Fig 1B1). We took advantage of the robust Gene Ontology annotations of the *S*. *cerevisiae* proteome to detect all significant and specific overrepresentations of one or more amino acids at position 2 in protein subsets defined by common GO annotations.

In a first step, we scanned the diverse GO terms describing the components, the cellular pathways or the protein molecular functions and we calculated the frequencies of the 20 amino acids at position 2 in the associated protein subsets. We then identified the subsets displaying significantly higher frequencies than expected by the overall distribution of amino acids at position 2. We assessed the significance of the enrichments by calculating the HGT scores derived from p-values obtained in hypergeometric tests. With this strategy, we identified 232 GO terms with a significantly increased frequency (HGT>4) for at least one amino acid. These GO terms were distributed as follows in the different GO categories: 72 GO terms corresponding to components, 138 to cellular pathways and 22 to molecular functions (S1 Table). We designed a dedicated algorithm to reduce this initial list and eliminate information redundancy in each category (See S1 Supplementary Methods and S2 Fig). Rather than relying on the hierarchical relationships between the GO terms, as usually done by existing solutions [28], our algorithm directly analyzes the characteristics of the protein subsets defined by these GO terms, such as the number of proteins, the enrichment factors, and the overlaps between the subsets. In a first filtering step, we eliminate GO terms with the lowest frequency bias (<1.8 fold change) and corresponding to very generic components or processes, which typically include a very high number of proteins. Our algorithm then retains the GO terms encompassing the largest number of proteins and maximizing the coverage of the original dataset (called BestN GO terms, see Methods). When possible, it complements this set of bestN GO terms with one or more smaller GO terms displaying the highest position 2 frequency biases (called BestF GO terms, see Methods). Each BestF is ultimately linked to the nearest BestN based on the overlap of their respective set of proteins.

Fig 1B2 and 1B3 illustrates the results of this filtering for the GO category "components" and represents the amino acid usage biases observed in the 17 GO terms that were finally retained after processing the initial list of 72 GO terms. Results for the other two GO categories, "cellular pathways" and "molecular functions", are available as supplementary data (see S1 Table and S3 Fig in which the original lists were reduced from 138 to 21 and from 22 to 10 GO terms, respectively). The final list of GO terms "components" includes the 6 BestN GO terms "Ribonucleoprotein complex", "Chromosome", "Mitochondrion", "Nuclear pore central transport channel", "Extracellular Region" and "Storage vacuole" (Fig 1B2). These GO terms covered 85% of the proteins with the amino acid preferences associated with the 55 GO terms retained after the first filtering step that removed low bias generic GO terms. The 11 GO BestF terms (Fig 1B3) provided a more detailed picture of protein subsets with specific N-terminal amino acid usage. For example, "90S preribosome" (3.4-fold increase in glycine utilization at position 2) and "large cytosolic ribosomal subunit" (5.1-fold increase in alanine utilization at position 2) led to a much better characterization of the proteins involved in the bias observed in the more general GO term "ribonucleoprotein complex". Similarly, the "Kinetochore" proteins (4.4-fold increase in aspartate utilization at position 2) explain a large part of the aspartate bias detected in the GO term "Chromosome" since it accounts for 12 of the 21 proteins responsible for this bias detection. The BestF GO terms also pointed to several mitochondrial sub-compartments with high preferences for leucine at position 2 (4.0- to 13.8-fold increase in leucine usage), suggesting that this bias involved only a specific subset of the mitochondrial proteins localized in the "Mitochondrial inner membrane” and the "Mitochondrial matrix".

The physiological significance of the amino acid utilization biases detected remains to be elucidated, and some of them might be related to the activity of N-terminal modifying enzymes such as MetAPs or NATs. One of the strongest biases revealed by our approach is the dramatic overrepresentation of leucine at position 2 in GO terms related to the mitochondrial compartment. In this work, we sought to clarify, in *S*. *cerevisiae*, the functional significance of usage bias at position 2 of mitochondrial precursors and its potential relationship with NatC.

### The N-terminal Mitochondrial Targeting Sequence has a specific conserved signature at position 2 typical of NatC potential substrates

**Fig 2:**
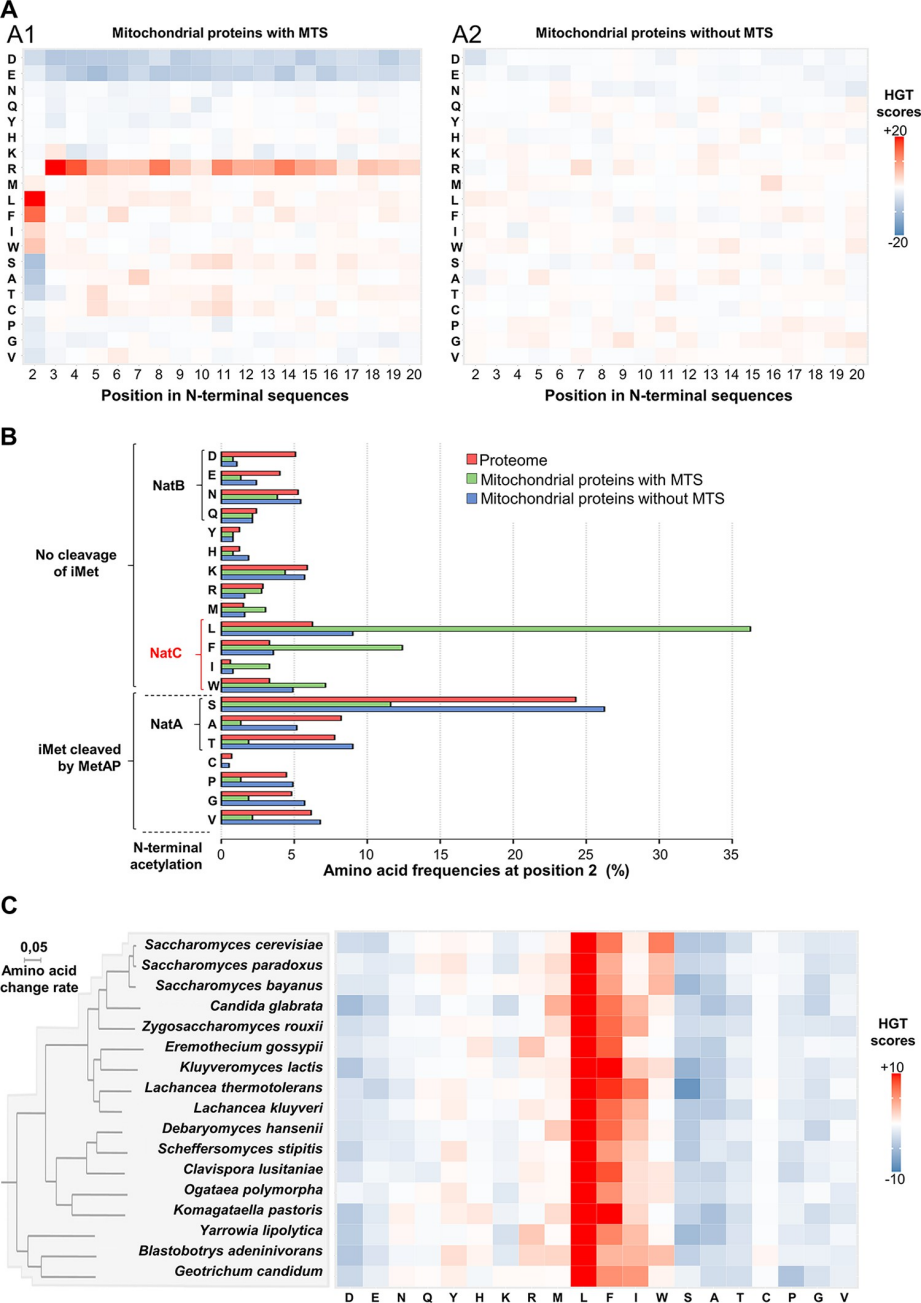
The specific N-terminal preference for large hydrophobic amino acid in MTSs make them putative substrate of NatC. (A) Heatmap of HGT scores showing specific uses of amino acids in MTSs, including the previously unknown specific preference at position 2 for large hydrophobic amino acids. HGT scores were calculated to compare amino acid usage at the first 20 positions of mitochondrial precursors with (A1) or without (A2) an MTS with their average usage in the proteome at these same positions. (B) Graphical representation of amino acid frequencies at position 2 of mitochondrial proteins revealing that a large majority of those carrying an MTS are putative substrates of NatC. The following amino acids are significantly overrepresented in position 2 of MTS compared to the proteome: Leu (p-val = 10^−71^), Phe (p-val = 10^−15^), Trp (p-val = 10^−6^) and Ile (p-val = 10^−4^). (C) Heatmap of HGT scores showing that amino acid preferences at position 2 of MTSs are conserved in 17 budding yeasts of the *Saccharomycotina* lineage. In each species, HGT scores were calculated to compare the use of amino acids at position 2 of MTSs with their average use at the same position in the corresponding proteome. The overrepresentation of large hydrophobic amino acids, especially leucine, is observed in all analyzed species. The phylogenetic tree was modified from (26).

We first investigated whether the high overrepresentation of leucine at position 2 of the mitochondrial precursors could correlate with other specific features in the amino acid composition of their N-terminal region. Especially, precursors of matrix, inner membrane and intermembrane space proteins are dependent for their mitochondrial import on an N-terminal sequence forming an amphiphilic alpha helix, 15–50 residues-long, enriched in hydrophobic and positively charged residues [29,30]. Such a mitochondrial targeting sequence, called MTS, is observed in 361 of the 726 yeast proteins annotated as mitochondrial with high confidence (S2 Table, see Methods). Consistently, the 361 mitochondrial precursors harboring a MTS showed a dramatic overrepresentation of arginine and an underrepresentation of negatively charged residues in the positions 3 to 20 of their N-terminal region (Fig 2A1). This profile contrasted strongly with that of mitochondrial precursors lacking MTS (Fig 2A2). Strikingly, we found that the bias at position 2 described above for the proteins associated with mitochondrial GO terms was specific of mitochondrial proteins with a MTS (Fig 2A1). More precisely, four hydrophobic residues (Leu, Phe, Trp and Ile) are significantly overrepresented (HGT value ranging from 3.9 for isoleucine to 71 for leucine) at position 2 of proteins with MTS (Fig 2A1). Furthermore, it should be noted that these biases in favor of Leu, Phe, Trp and Ile are strictly restricted to position 2 of the MTS.

Quantitative analysis of the distribution of amino acids at position 2 of the MTSs (Fig 2B) shows the importance of this previously unknown N-terminal signature of the mitochondrial addressing sequence: nearly 60% of them have an N-terminal signature with a Leu, Phe, Trp and Ile, which contrasts strongly with the low representation of the latter at position 2 in the proteome (see L, F, I, W residues frequencies in Fig 1A). For instance, leucine accounts for more than 35% of the amino acids observed at position 2 of the MTSs, whereas it is poorly used in the *S*. *cerevisiae* proteome at this position (Fig 1A). Interestingly, we also found an overall significant under-representation at position 2 of the MTSs of amino acids with small radii of gyration (Gly, Ala, Ser, Cys, Thr, Pro and Val), known to induce the cleavage of iMet by methionine aminopeptidase [6]. Finally, the analysis of amino acid usage biases in the MTSs of 17 budding yeasts of the *Saccharomycotina* lineage [26,27], demonstrated that this specific signature at position 2 is a conserved characteristic of mitochondrial addressing sequences (Fig 2C). Indeed, in all the studied species, we observed: (1) a strong over-representation of the same hydrophobic amino acids at position 2 and in particular of leucine whose HGT score exceeds the value of 20 in all species, and (2) a bias restricted to position 2 since the HGT scores of leucine, phenylalanine, isoleucine and tryptophan were systematically lower than 3.9 for all the other positions of the MTSs (S1B Fig).

Interestingly, the conserved amino acid bias described above at position 2 of MTSs perfectly correlated with the known specificities of N-acetyl transferases in yeast (Fig 2B). Indeed, leucine, phenylalanine, isoleucine and tryptophan, which are overrepresented at this position, are known to define potential substrates of NatC in yeast when positioned after iMet [9]. Conversely, serine, alanine and threonine, which are the most under-represented at this position, are known to induce iMet cleavage and subsequent targeting by NatA [8].

Together, these observations strongly suggest that position 2 of N-terminal mitochondrial targeting sequences is under selective pressure and that the nature of the residue at position 2 is crucial for the fate of mitochondrial precursors imported through the MTS-dependent pathway. The correspondence between the amino acid usage bias at position 2 of MTSs and the preferences of NatC suggests a link between mitochondrial precursors fate and NatC targeting. They raised two important questions addressed in the following sections. First, are mitochondrial precursors indeed targeted by the N-terminal acetyl transferase NatC? And second, what are the functional consequences of changing the residue located at position 2 of a typical MTS?

### Selective Translating Ribosome Affinity Purification reveals co-translational targeting by NatC of mitochondrial precursors harboring the identified N-terminal signature

Our current knowledge of the substrate specificities of NatC in yeast identifies proteins with ML-, MF-, MI- and MW- N-termini (i.e. iMet followed by Leu, Phe, Ile, or Trp) as its potential substrates. It was deduced from the observation of the loss of N-terminal acetylation of various iso-1 cytochrome c reporter proteins mutated at position 2 in yeast strains lacking NatC, which was further confirmed for a small subset of cellular proteins [9,31–33]. Partial N-terminal acetylome of *S*. *cerevisiae* were more recently obtained [8,10,34] that covered up to 10% of the proteome, corresponding to the most abundant cellular proteins. Unfortunately, these data included very few NatC potential substrates (between 0 and 60 for each NatC typical N-termini). Recently published data describing globally the *in vivo* substrates of NatC by studying the impact of NatC depletion on the yeast N-terminal acetylome detected 57 NatC substrates among which only 8 were mitochondrial proteins [12]. However, inactivation of NatC in *S*. *cerevisiae* has been consistently reported to induce growth defects on nonfermentable carbon sources, such as glycerol and ethanol (32–36). We also found that in the absence of NatC, yeast cells were much more sensitive to the mitochondrial ATP synthase inhibitor oligomycin, with almost complete loss of viability when grown on glycerol medium in the presence of a low growth-limiting concentration of this drug (S4A Fig). Based on the use of amino acids at position 2, our *in-silico* analysis predicted that approximately 200 mitochondrial precursors could be acetylated by NatC, which could provide a molecular explanation for the mitochondrial defects observed in cells lacking NatC, including their greater reliance on mitochondrial ATP production.

**Fig 3:**
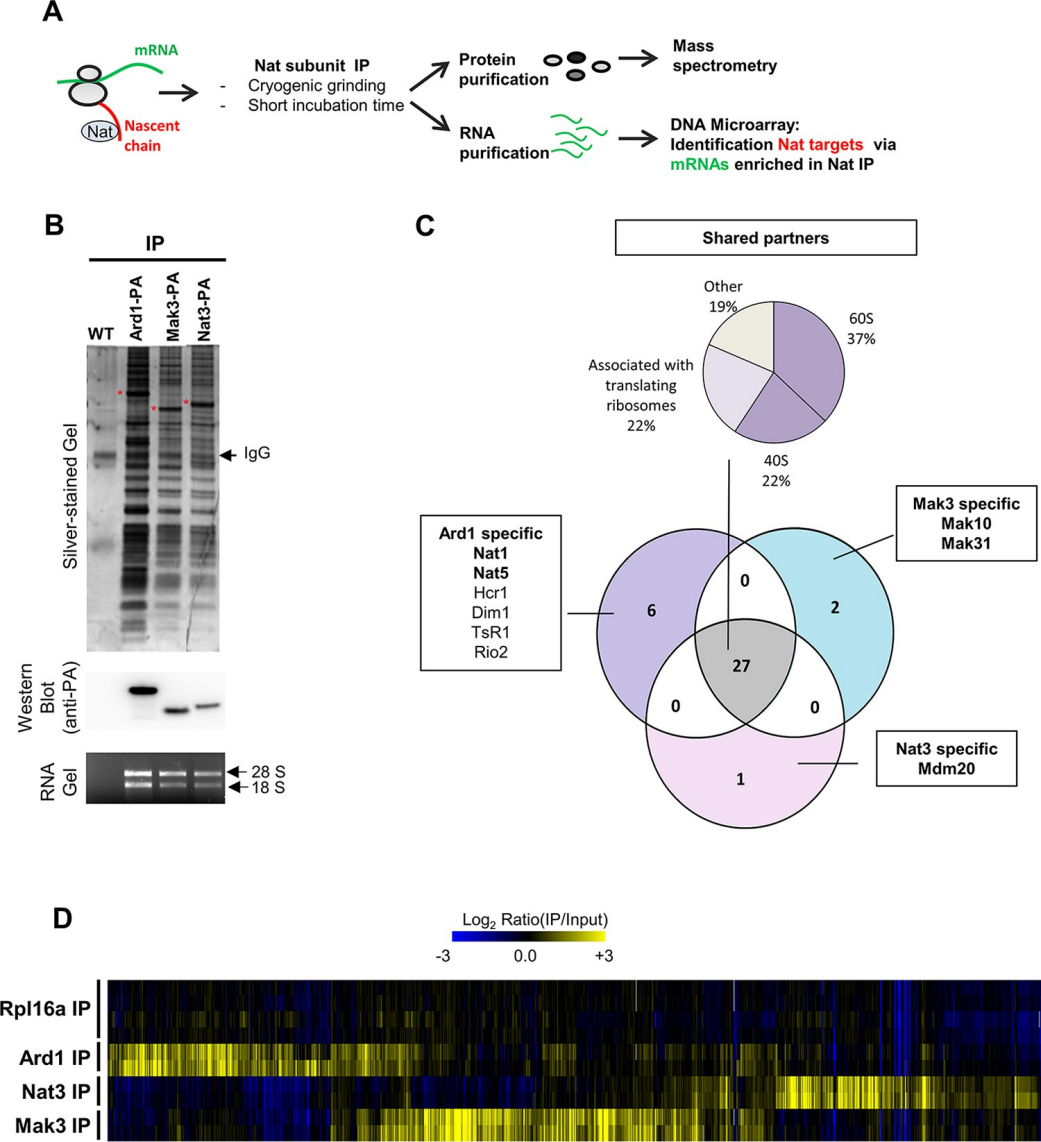
Capture of cotranslational targets of NatA, NatB and NatC by Selective Translating Ribosome Affinity Purification. (A) Sel-TRAP (Selective Translating Ribosome Affinity Purification) experimental approach. Immunoprecipitations of NatA, NatB and NatC were performed on cells expressing respectively Ard1-PA, Nat3-PA and Mak3-PA. Control experiments was also performed with an untagged strain (WT). Protein (B and C) and RNA (B and D) contents were analyzed from immunoprecipitated samples (IP). (B) Top panel: SDS-PAGE followed by silver staining of immunoprecipitated protein fractions from untagged or PA fusion protein expressing-cells. The position of the Ard1-PA, Nat3-PA and Mak3-PA fusion and IgG are indicated by red stars and a black arrow, respectively. Middle panel: Western blot detection of PA fusion proteins in the same samples. Bottom panel: Agarose gel electrophoresis and ethidium bromide detection of 28S and 18S rRNA in immunoprecipitated RNA fractions. (C) Comparison of the composition of NatA (Ard1p), NatB (Nat3p) and NatC (Mak3p)-associated ribosomes by mass spectrometry analysis of immunoprecipitations protein fractions. Venn Diagram represents the overlap of the three different sets of co-immunoprecipitated protein partners: for each sector the number and the name of interactants reproducibly detected in two independent experiments are indicated (S4 Table). Partners indicated in bold correspond to the known subunits of NatA, NatB and NatC complexes. The pie chart shows the functional distribution of proteins for the shared partners in the three datasets. (D) Microarray analysis of mRNA contents from total extract (Input) and immunoprecipitated fractions (IP) identified mRNAs specifically enriched after sel-TRAP of NatA, NatB and NatC. The mRNA enrichment values, defined as log2(Ratio) IP/input, were calculated after hybridization of reverse transcribed labeled cDNA. Immunoprecipitated mRNAs from cells expressing Rpl16A-PA are used to obtain reference enrichment values in the canonical set of translating ribosomes. Hierarchically clustered heatmap represents enrichment value for mRNAs enriched, in two independent experiments, in at least one of the NAT immunoprecipitations relative to that of Rpl16Ap (Enrichment value corrected with Rpl16A≥0.8, see S3 Table). Each line represents an experiment, each column a gene.

Mitochondrial precursors steady state levels are extremely low, since, after their synthesis, they are quickly imported in mitochondria where their N-terminal addressing sequence is cleaved [29]. Hence, detecting them with protein-based approaches is challenging. To overcome this limitation, we decided to use the Selective Affinity Purification of Translating Ribosomes (sel-TRAP) method, to capture by immunoprecipitation the various NatC-targeted nascent chains, as well as their associated mRNAs, which we characterized using transcriptomic analyses (Fig 3A, see also microarray data in S3 Table). This approach had already proven sufficient sensitivity to study the selectivity of various nascent chain associated factors [35–38]. We verified that the addition of a protein A tag at the C-terminus of Mak3p, which is required for the sel-TRAP experiment, did not disrupt cell growth on glycerol medium, indicating that NatC is still catalytically active in the tagged strain (S4B Fig). To validate our strategy, we also conducted sel-TRAP experiments on NatA and NatB, which preferences were previously confirmed by studying the global impact of their deletion on N-terminal acetylome [8–11]. Hence, we immunoprecipitated protein A-tagged versions of the catalytic subunits of each of the three NATs, Ard1p (NatA), Nat3p (NatB), and Mak3p (NatC) (Fig 3B, top and middle panel). The detection of rRNAs in the immunoprecipitated fractions (Fig 3B, bottom panel) and the analysis of the protein fractions of the three immunoprecipitations compared with the mock experiment (Fig 3C, see also proteomic data in S4 Table) confirmed that we had purified translating ribosomes that were mostly distinct by the specific presence of the catalytic and accessory subunits of the immunoprecipitated N-terminal acetyltransferase complexes. Transcriptomic analyses of the immunoprecipitated samples compared to the input RNAs identified sets of enriched mRNAs likely encoding the respective substrates of each NAT (Fig 3D, see also transcriptomic data in S3 Table). As expected from the literature, these sets of potential targets for NatA, NatB and NatC were largely non-overlapping, with few mRNAs being enriched in more than one condition. We also analyzed mRNA co-immunoprecipitated with the canonical Rpl16A ribosomal protein, which confirmed the specificity of the mRNAs identified in the NATs sel-TRAP experiments (Fig 3D).

To verify that the NATs sel-TRAPs have specifically purified their respective substrates, we performed a threshold-independent analysis of the proportion of amino acids encoded at ORFs position 2 in the transcriptomic data. We detected a specific and significant increase in serine in the NatA immunoprecipitation experiment, aspartate in that of NatB, and leucine and phenylalanine in that of NatC (S5 Fig). These results are globally consistent with the known substrate specificity of the three NATs in *S*. *cerevisiae* (see Introduction). However, all the expected N-termini were not detected as enriched, which could reflect differences in the efficiency of recognition of their potential substrates by the NATs. Consistent with this idea, N-terminal acetylome data has previously revealed that, in many cases, only a fraction of NATs expected substrates is actually acetylated [8,10,34]. For example, among the proteins potentially targeted by NatA, almost all of those with a Ser- N-termini were acetylated, whereas only about 50% of those with Ala- or Thr- N-termini were, and almost none of the other potential NatA targets (Pro-, Gly-, and Val- N-termini). Similarly, most of the few NatC potential substrates detected in N-terminal acetylome data were only partially acetylated. It should also be kept in mind that sel-TRAP technology captures co-translational targets that co-purify with the immunoprecipitated NATs and that the transient nature of enzyme-substrate connections could limit the detection of the weakest interactions.

**Fig 4:**
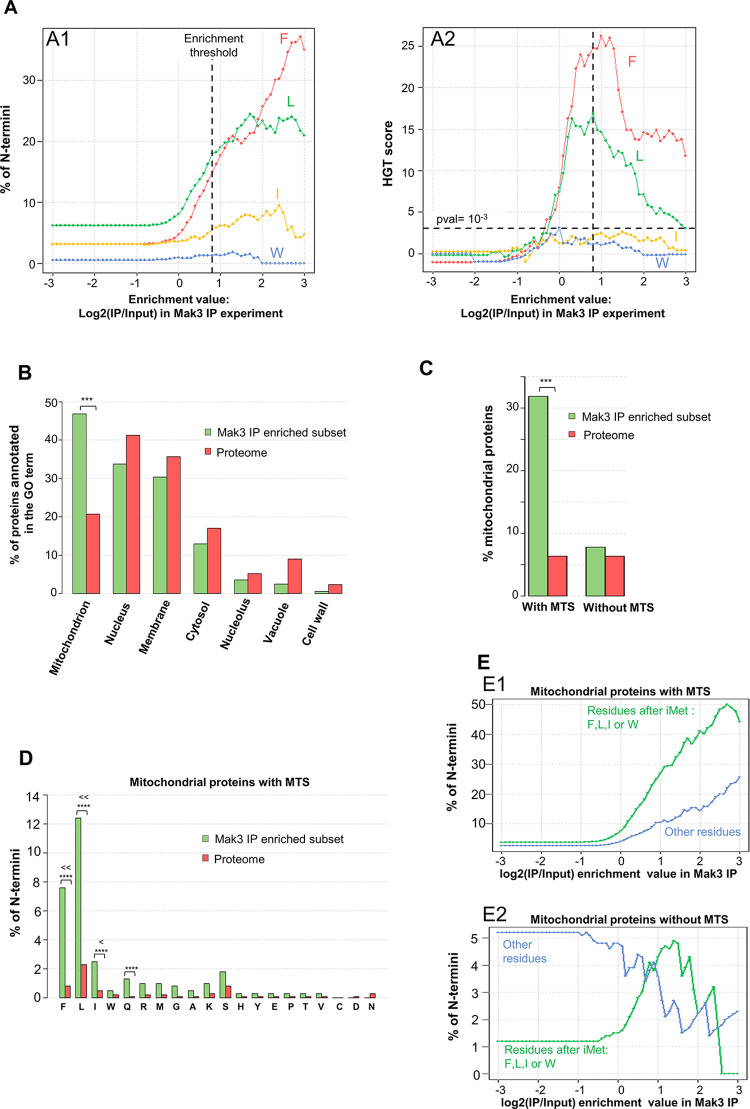
Sel-TRAP confirms that mitochondrial precursors with MTS are the predominant NatC targets. (A) Threshold-independent analysis of the proportion of N-termini in proteins encoded in the transcriptomic data from Mak3-PA immunoprecipitation confirmed that sel-TRAP experiments have specifically purified canonical NatC substrates. The proportion of residues defining putative NatC substrates (i.e. F, L, I, or W at position 2) as a function of enrichment value is shown in A1: for each x-axis value, the y-value shown is the % of N-termini among those encoded by mRNAs with an enrichment value greater than or equal to the x value. Statistical evaluation of the observed increases was performed by HGT score analyses (A2), which confirmed a significant enrichment of the MF and ML N-termini in the most enriched protein set. The horizontal dashed line in A2 indicates the threshold for significance of the HGT score (p-value equal to 10^−3^). The vertical dashed line in A1 and A2 indicates the mRNA enrichment threshold value of 0.8 chosen to define the set of putative NatC targets detected by the sel-TRAP approach. (B) Distribution of proteins of the diverse cellular compartment among NatC putative targets showing the high selectivity of NatC for mitochondrial precursors, which represent 47% of the putative targets identified versus 21% for all cellular proteins (p-value = 10^−33^). No enrichment among putative NatC targets was detected for proteins with GO annotation corresponding to other cellular compartments. (C) Distribution of MTSs among proteins identified as NatC putative targets revealing that 32% of them have a MTS versus 6% in the proteome (p-value = 10^−54^). No significant enrichment of the mitochondrial precursors without MTS was detected in the sel-TRAP data. (D) Distribution of different N-termini in MTS identified in putative NatC targets showing strong enrichments for residues defining NatC canonical substrates (i.e. F, L, I, W at position 2) but also unexpected enrichment for other N-termini. Significant enrichment was detected for MF, ML, and MI N-termini (p-values equal to 10^−24^, 10^−23^, and 10^−25^, respectively) and, to a lesser extent, for MQ N-termini (p-value equal to 10^−4^). Stars indicate significant p-values (p-value≤10^−4^). (E) Threshold-independent analysis of the proportion of N-termini, defined as the amino acid observed at position 2, in mitochondrial precursors detected in sel-TRAP data confirming non-canonical enrichment among precursors with MTS (E1). The proportion of MTS with residue after iMet that do not define them as putative NatC substrates rise from 2.6% in the proteome to 25% in the most enriched proteins in sel-TRAP data (enrichment value ≥3). This increase was not detected among mitochondrial precursors lacking MTS (E2).

While previous N-terminal acetylome data in absence of NatC reported only few NatC substrates [12], sel-TRAP allowed for the first time a global study of NatC targets *in vivo* in *S*. *cerevisiae*. After immunoprecipitation of Mak3p, we observed in our threshold-independent analysis a significant increase specific to leucine and phenylalanine at position 2 of the proteins encoded by the most enriched mRNAs (green and red curves in Fig 4A1, see also S5C Fig). The significance of these increases was confirmed by the observation that the HGT scores for the ML- and MF- N-termini reached a maximum value near 15 and 25, respectively (green and red curves in Fig 4A2). This is consistent with the known substrate specificity of NatC and suggests more efficient co-translational acetylation of the initiator methionine when followed by these residues rather than by isoleucine or tryptophan. Furthermore, in agreement with our prediction, nearly 50% of the enriched mRNAs (enrichment threshold value equal to 0.8) encoded mitochondrial precursors (Fig 4B) and only those imported through the MTS-dependent pathway were observed enriched in the sel-TRAP data (Fig 4C). Among these precursors, the most significant enrichments were observed for those with a leucine (HGT = 24), phenylalanine (HGT = 23), or isoleucine (HGT = 5) at position 2 of their mitochondrial addressing sequence (Fig 4D).

Intriguingly, we also observed in the sel-TRAP data a slight enrichment of MTSs without a typical signature of NatC targets at position 2 (amino acid other than F, L, I, W in Fig 4D). With the exception of glutamine at position 2 (HGT = 4), these enrichments were not significant, but this could be due to the low number of precursors with a given amino acid at position 2 of their MTSs sequence (between 3 and 42). Indeed, a threshold-independent analysis showed that the enrichment of mitochondrial precursors with MTS was mainly related to their N-termini that define them as potential NatC substrates (F, L, l, W amino acid at position 2, green curve in Fig 4E1) but that MTSs lacking this signature at position 2 contributed significantly to MTSs enrichment in the sel-TRAP data (blue curve in Fig 4E1). This unexpected enrichment was not observed for mitochondrial precursors lacking MTS (blue curve in Fig 4E2), as well as in the overall analysis of NatC sel-TRAP results (S5C Fig). It is unlikely that these enriched mitochondrial precursors lacking hydrophobic residues at position 2 are new putative substrates for NatC. Instead, we propose that the common intrinsic features of all MTS, i.e. their amphipathic nature, may be involved in interactions with NatC not necessarily involving its catalytic site.

Finally, sel-TRAP analysis clearly demonstrated that mitochondrial proteins with an N-terminal MTS are co-translationally recognized by NatC mainly when they have a leucine, a phenylalanine or to a lesser extent an isoleucine at position 2. Furthermore, unexpectedly, the observation that MTSs with other amino acids at position 2 were selectively enriched after NatC immunoprecipitation strongly suggests that other features of MTSs might be involved in their recognition by NatC.

### Substitution of leucine 2 by amino acids not overrepresented at position 2 of MTSs result in precursor accumulation and rescue of the dominant negative effect of an HSP60-13myc transgene

**Fig 5:**
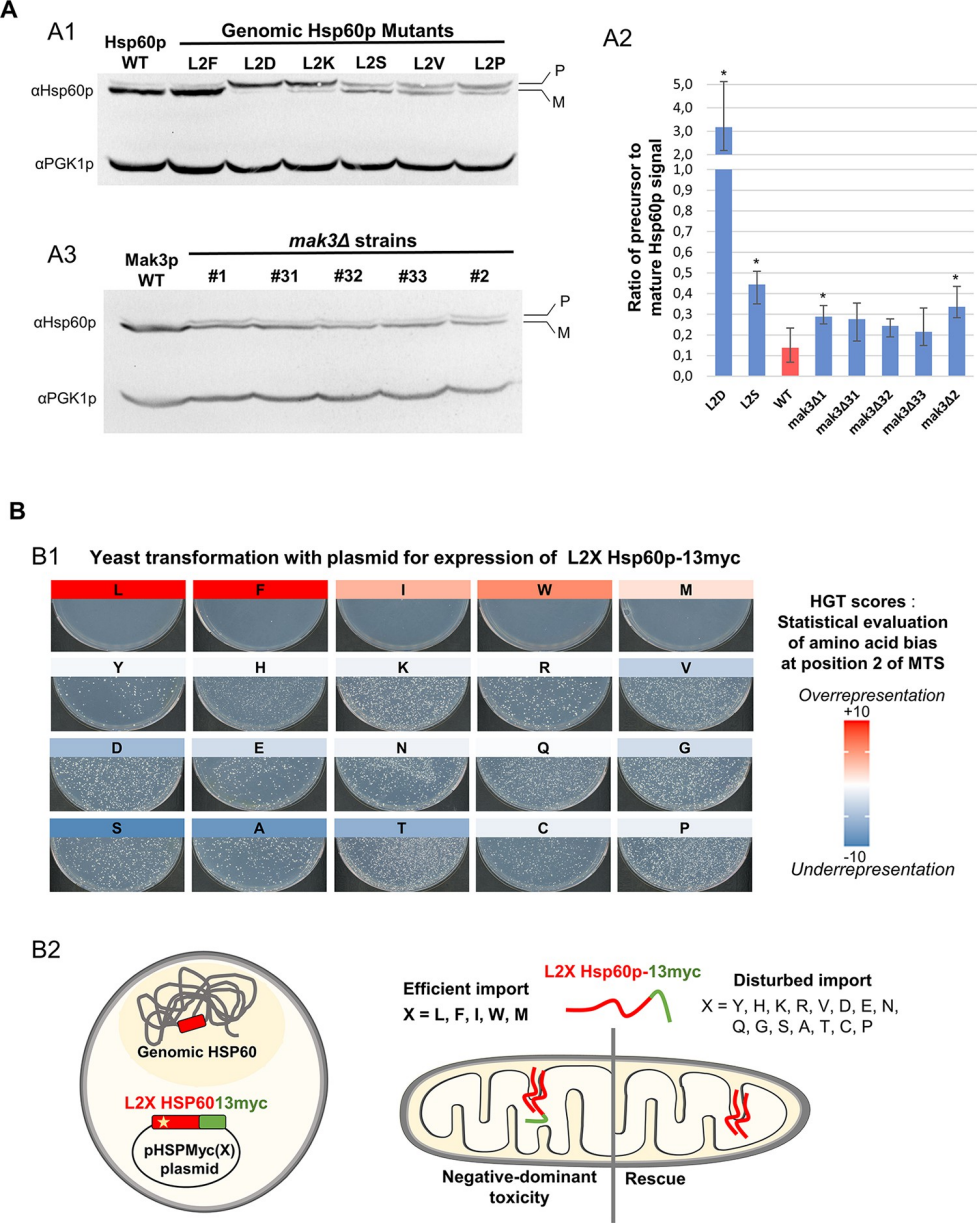
The critical role of large hydrophobic residues at position 2 of the MTS for efficient mitochondrial import of Hsp60p highlighted by mutagenesis. (A) Western blot analysis of protein extracts showing accumulation of Hsp60p precursor in L2X mutants (A1) or in NatC-deficient (*mak3Δ*) strains (A3). Panel A2 shows the quantification of the Hsp60p precursor/mature form ratio for L2D, L2S and five *mak3Δ* mutants (see also S5 Table). Precursor accumulation was clearly and reproducibly detected in L2D, L2K, L2S, L2V, L2P mutants (A1, see also quantification of the image shown in S6 Fig). The Hsp60p precursor band was slightly accumulated in all five *mak3Δ* strains analyzed (A3). The average ratio of precursor to mature Hsp60p signal (A2) ranges from 0.21 to 0.34 falling between the value observed in the unmutated strain (0.14) and that observed in the L2S mutant (0.44). These ratios were measured using western blot images obtained from protein extracts from three independent biological cultures. Stars indicate significant accumulation of the Hsp60p precursor in the mutant strains compared with the reference strain (one-tailed t test p-value ≤5%). (B) Leucine 2 replacement in the HSP60-13myc transgene can rescue its dominant lethality by preventing efficient import of the expressed mitochondrial precursor. Photographs of transformation plates (B1) showing that only a few clones are obtained after transformation of yeast with pHSPmyc(X) plasmids encoding Hsp60p-13myc versions with a large hydrophobic amino acid in position 2 of its MTS (X = Leu, Phe, Ile, Trp or Met). By contrast, transformations with plasmids expressing all other L2X mutants of Hsp60p-13myc resulted in more than 1000 transformants. This rescue of the toxicity of the Hsp60p-13myc transgene was interpreted as the consequence of an impaired mitochondrial import of the expressed precursor (B2), a conclusion supported by the observation that L2D mutation lead to cytoplasmic accumulation of Hsp60p precursor (see S7 Fig). The color scale representing HGT scores quantifying amino acid utilization bias at position 2 of MTSs (see also Fig 2A1) indicates that the amino acids that allow efficient import of Hsp60p, revealed by the lethality of the Hsp60-13myc transgene, are those that have been positively selected during evolution.

Finally, we explored the functional significance of selecting during evolution specific amino acids at the N-terminus of the mitochondrial addressing sequences. To this aim, we set up a mutagenesis experiment at position 2 of a typical MTS. We chose Hsp60p, an essential chaperonin of the mitochondrial matrix [39] as a model of the MTS-dependent import pathway because of (1) the presence of a highly conserved leucine at position 2 of its MTS (leucine 2 is observed in 13 of 17 *Saccharomycotina* yeasts studied) and (2) the availability of an antibody allowing sufficiently sensitive detection of its precursor when its mitochondrial import is impaired. In addition, to improve detection of Hsp60p precursors, we used the thermosensitive strain YPH499 pam16Δ-MAGN76D [40], which, even at the permissive temperature, exhibits a slight defect in mitochondrial import as evidenced by the detection of a low-intensity band corresponding to the precursor form of Hsp60p (Fig 5A1, Hsp60p WT lane). We used CRISPR/CAS9 technology to mutagenize this strain and create L2X genomic mutants of Hsp60p in which the leucine at position 2 was replaced by another amino acid (Fig 5A1, lanes corresponding to L2X mutants). Replacing leucine 2 in Hsp60p with aspartate, lysine, serine, valine or proline (Fig 5A L2D, L2K, L2S, L2V, L2P lanes) resulted in a dramatic increase in the intensity of the band corresponding to the Hsp60p precursor compared to that corresponding to its mature form. In contrast, substitution of leucine with a phenylalanine, another hydrophobic amino acid over-represented at position 2 of the MTS-containing protein group, had no impact on the Hsp60p profile observed by western blot (Fig 5A1, L2F lane). Quantification of the images (see Figs S6 and 5A2) strongly suggests an alteration in mitochondrial import of Hsp60p protein, with the ratio of precursor to mature signal increasing from 0.10 for the wild type and L2F Hsp60p mutant to a value ranging from 0.47 (L2S mutant) to 3.2 (L2D mutant) in the other mutant strains. Cellular fractionations confirmed this interpretation since, whereas the mature Hsp60p protein in the wild type strain was strongly enriched in the mitochondrial fraction, the Hsp60 precursor band that accumulated in the L2D mutant was detected only in the cytosolic fraction (S7 Fig).

We sought to determine whether the accumulation of Hsp60 precursors observed in the L2X mutant was due to a loss of N-terminal acetylation by NatC. We constructed five independent NatC mutants derived from the YPH499 pam16Δ-MAGN76D strain by replacing the MAK3 gene encoding the catalytic subunit of NatC with a KanR deletion cassette. For each of these 5 mutants, we could detect a slight accumulation of the Hsp60p precursor. However, as with their parental strain, this accumulation of precursor was close to the limit of detection of the western blot and was not consistently observed (Fig 5A3). We quantified the ratio of precursor to mature signal from three protein extracts from independent cultures (Fig 5A2). We also included three independent biological replicates from the L2D and L2S Hsp60p mutants in these western blot experiments, as references for strains in which an accumulation of precursor had been consistently observed. This quantitative approach confirmed a two- to threefold increase in the mean ratio of precursor Hsp60p signal to mature Hsp60p signal in Mak3-deleted strains compared to their parental strain (Fig 5A2). However, this precursor accumulation was only statistically significant for two of the mutant strains studied, with a one-tailed t-test p-value close to 2.5% (2.7% and 2.3% for *mak3Δ* clones 1 and 2, respectively). Moreover, this effect was definitely not comparable to what we observed in L2X mutants (the p-value for the L2S mutant with the lowest precursor accumulation being equal to 0.4%). This result indicates that NatC plays only a minor role in the dependence of mitochondrial import of Hsp60p on its N-terminal residue, and prompted us to characterize the set of amino acids at position 2 of its MTS that were competent for optimal mitochondrial targeting.

In a next step, we developed a screen to easily test the effect of all possible amino acid substitutions at position 2 of Hsp60p. For this purpose, we took advantage of an HSP60-13myc allele that we found to have a dominant negative effect. Indeed, the transformation of *S*. *cerevisiae* with the pHSPmyc(L) plasmid, which allows the expression of the HSP60-13myc allele, led to no colony (plate L in Fig 5B1). To interpret this result, we hypothesized that the 13-myc C-terminal extension abolished the activity of Hsp60p C-terminal domain, previously shown to play a crucial role for the function of the protein [41,42]. Thus, since Hsp60p is essential for cell survival, we attributed the near absence of clones after transformation with pHSPmyc(L) to a dominant negative effect of the myc-tagged version of Hsp60p on the endogenous wild-type form. After being imported in mitochondria, Hsp60p forms a tetradecameric chaperonin complex in the mitochondrial matrix required for its own assembly [41]. Thus, the association of the deficient protein Hsp60p-13myc with endogenous wild-type Hsp60p would lead to the inactivation of the Hsp60p complexes and the observed drastic loss of viability (Fig 5B2). We further observed that an altered mitochondrial import of Hsp60p, as induced by the cis mutations L2D, L2K, L2S, L2V, L2P (Fig 5A1), resolves the dominant negative effect of the 13-myc tag. More than 1,000 transformants were obtained with the pHSPmyc(X) plasmids corresponding to these mutations, where X represents the amino acid that replaces leucine at position 2 of Hsp60 in the Hsp60p-13myc protein (Fig 5B1).

Strikingly, after testing all possible amino acids at position 2 of Hsp60p-13myc, we found that the two groups of amino acids defined on the basis of their ability to rescue C-terminal tag toxicity correlated strongly with their enrichment at position 2 of *S*. *cerevisiae* mitochondrial addressing sequences, as assessed by our statistical analyses (Figs 2A1 and 5B1). Indeed, the amino acids that did not result in more than 1–3 clones after transformation were those we showed to be to be enriched at position 2 of MTSs, i.e., leucine (HGT = 71), phenylalanine (HGT = 15), isoleucine (HGT = 4), tryptophan (HGT = 6), and methionine (HGT = 1.6). Western blot analysis revealed that the few clones obtained for these 4 amino acid substitutions did not express Hsp60p-13myc protein (S8A Fig) and likely escaped toxicity through plasmid mutations, which was confirmed by sequencing some of these plasmids. For all other amino acids (except tyrosine which showed a lower number of transformants), we observed more than 1,000 colonies after transformation by pHSPmyc(X) plasmids. Western blot analysis of the resulting clones confirmed that they all express Hsp60p-13myc protein at a level roughly comparable to that of the endogenous protein (S8B Fig). The only exceptions are the clones transformed with pHSPmyc(Y) in which the level of Hsp60-13myc protein was systematically reduced (S8 Fig). In addition, we consistently observed, in all clones with a rescue phenotype, an Hsp60p-myc band corresponding predominantly to the non-imported precursor form (see for example in S8B Fig the profile of the L2Q mutant for which both bands are visualized with the anti-myc antibody). More surprisingly, we also observed a defect in import and/or processing of the endogenous, wild type, Hsp60p, reflected by the clearly visible band corresponding to the precursor form of the protein (S8B Fig). This observation may indicate that a small fraction of the Hsp60p-13myc proteins may have been imported, resulting in a significant, although not lethal, loss of Hsp60p function that could be associated with impaired processing of mitochondria-targeted endogenous Hsp60p precursors, as previously observed upon inactivation of mitochondrial chaperonin [39,43].

The results obtained in our mutagenesis experiments allowed us to demonstrate the dramatic functional consequences of modifying the residue located at position 2 of the mitochondrial addressing sequence. These results, which fully validated our *in-silico* predictions, also supported the existence of strong evolutionary constraints that shaped the current amino acid preferences at position 2 of MTSs, thus ensuring an optimized mitochondrial import.

## Discussion

In this work, we developed an *in-silico* approach to identify functional groups of proteins with N-terminal amino acid usage biases. The resulting functional mapping of N-terminal residues reveals possible evolutionary constraints at position 2 of nascent chains and highlights potential early mechanisms of protein regulation or targeting. In particular, we discovered the potential critical role of the residue located at position 2 of mitochondrial N-terminal targeting sequences in *S*. *cerevisiae*. This prediction was experimentally validated using an original system based on a dominant-negative allele of HSP60, that we set up to test the efficiency of mitochondrial import after mutagenesis at position 2 of the MTS. Strikingly, we observed that only the 5 hydrophobic amino acids (Leu, Phe, Ile, Trp and Met) over-represented at this position allowed efficient mitochondrial import of Hsp60p protein. This finding raises the question of the nature of selective pressures acting specifically on position 2 of the MTSs in the context of their other well-established characteristics. It also strongly suggests the involvement in optimizing mitochondrial precursor import of N-terminal modification enzymes, including NatC, which we found to co-translationally interact with MTS-bearing precursors.

### The N-terminal signature of MTSs in light of the current knowledge about mitochondrial import mechanisms

MTSs are typically 15–50 amino acid residues in length and form positively charged amphipathic α-helices. It has been suggested that these sequences possess several biochemical features involved in their interaction with binding sites of the mitochondrial translocation machinery [29]. First, the amphipathic alpha helix interacts with two outer membrane translocase (TOM) receptors: the hydrophobic surface is recognized by Tom20 and the positively charged one by Tom22 [44–46]. In addition, translocation of the precursor into mitochondria can occur via an acidic binding chain involving the interactions between MTSs basic residues and some residues from Tom7p, Tom22p, and Tim23p [47]. Finally, the mitochondrial membrane potential (Δψ) generates an electrophoretic force on the MTSs and the net positive charge of the MTSs is the critical determinant of this effect [48,49]. Taken together, these data indicate that the hydrophobicity of MTSs is mainly determinant for their primary interaction with the outer mitochondrial membrane receptor Tom20p.

In addition, TargetP2.0, a new N-terminal sorting signal detection tool based on a machine learning approach, has recently further improved the prediction of mitochondrial presequences [50]. By analyzing the attention layer of their final network, these authors found that the residue at position 2 of the presequences had a strong influence on their classification. More specifically, they observed that, in fungi, peptides targeted to mitochondria and to the endoplasmic reticulum (ER) showed at position 2 an underrepresentation of residues that allow the removal of the initiator methionine, i.e. Ala, Cys, Gly, Ser, Thr, or Val [50]. This observation is consistent with the results we obtained in *S*. *cerevisiae* (Fig 2B). Indeed, we found that only 20% of MTSs have amino acids at position 2 allowing the removal of their iMet compared to 56% within the proteome and 69% among cytosolic proteins. We also detected amino acid preferences at position 2 of proteins excreted reflecting the characteristics of their N-terminal ER signal peptide, including the previously described under representation of Ser, Ala, Gly and Thr at position 2 (see GO category “extracellular region” in Fig 1B2). Of note, Forte et al. also previously observed that less than 25% of ER signal sequences would be predicted to be MetAPs substrates and further demonstrated that removal of iMet from carboxypeptidase Y precursor inhibits its ER translocation [51]. Similarly, we can speculate based on our results and on the observation made by Almagro Armenteros et al. that removal of iMet may be deleterious to mitochondrial import.

In favor of this hypothesis, we observed that the optimal residues at position 2 of MTSs, i.e. Leu, Phe, Ile, Trp and Met, have high hydrophobicity indices, but also that none of them allow iMet removal. Conversely, the small residues Ala and Cys, which are also highly hydrophobic but induce iMet cleavage when positioned at position 2 of the nascent chains, are underrepresented at this position of the yeast MTSs. Therefore, the N-terminus of most MTSs will be characterized by the sequence M(L/F/I/W/M) with two consecutive hydrophobic residues in positions 1 and 2 and this hydrophobic N-terminus may be important in the early binding of mitochondrial precursors to the mitochondrial surface. Indeed, several analyses indicate that consecutive hydrophobic residues could be involved in the binding of MTSs to Tom20p. In particular, by measuring the binding of a peptide library to Tom20, a consensus sequence θφχβφφ was identified (where θ, β, φ, and χ represent a hydrophilic, basic, hydrophobic, and any residue, respectively) and proposed as a Tom20 binding motif [52]. Bioinformatic analysis extended this consensus by identifying statistically significantly enriched hexamers in the 30 first residues of MTSs, and their detection was implemented in MitoFates, one of the most efficient tools for MTSs prediction to date [53]. Most of these hexamers showed strong potential to form amphiphilic helices and includes several consecutive hydrophobic residues.

**Fig 6:**
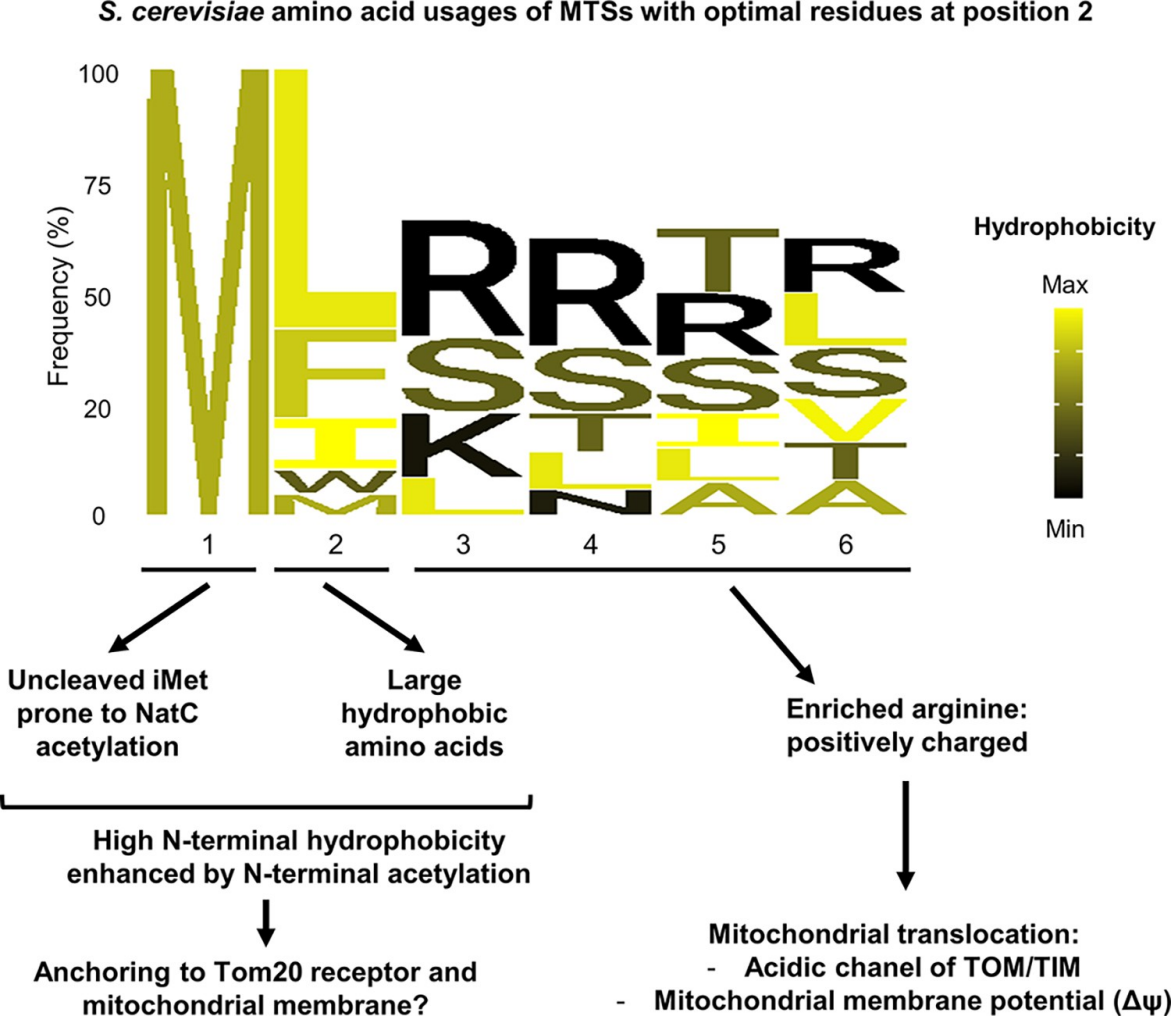
Model explaining why large hydrophobic residues were selected at position 2 of MTSs. Probably due to high selective pressure, more than 60% of *S*. *cerevisiae* MTSs have a large hydrophobic residue (Leu, Phe, Ile, Trp, Met) in position 2. The amino acids observed in this class of MTSs are represented by a sequence logo [54] in which, for position 3 to 6, only the most frequent residues representing at least 60% of the observed residues are displayed. Due to their large hydrophobic residue at position 2, these MTSs are characterized by a high N-terminal hydrophobic signature, contributed by their residues at positions 1 and 2, and virtually enhanced by NatC’s N-terminal acelylation of their uncleaved iMet. We propose that this N-terminal hydrophobicity was selected during evolution because it provided a favorable environment for the interaction of MTSs with the mitochondrial membrane and the Tom20p receptor. Furthermore, we postulate that the identity of the residue at position 2 influences the overall distribution of amino acid profiles in MTSs and enabled the observed high enrichment of arginine required for MTS translocation into mitochondria. This configuration of residues in MTS is likely to promote efficient mitochondrial import with limited interference between the two driving forces, hydrophobicity and positive charge, required in this mechanism.

By integrating these data from the literature and our results on MTS position 2, we propose a model explaining the critical role of hydrophobic residues at the N-terminus of mitochondrial addressing sequences (Fig 6). We can hypothesize that the selection of large hydrophobic amino acids at position 2 of MTSs was a successful evolutionary solution, the consequences of which were high N-terminal hydrophobicity due to (1) conservation of the hydrophobic iMet, and (2) possible N-terminal neutralization of the N-terminal amine by acetylation by NatC. Positioning the hydrophobic site directly at the N-terminus of MTS would then allow for early interaction with Tom20p and the mitochondrial membrane. Consequently, the arginines that provide the positive charge necessary for translocation of the precursors to mitochondria could be highly enriched from residue 3 of MTS, without compromising the interaction with Tom20p.

### A conserved role of the N-terminal signature in the mitochondrial import of MTS-bearing proteins

In support to our observation of significant biases at position 2 of the MTS in yeast, our mutagenesis experiments using Hsp60p as a model revealed the importance for proper mitochondrial import of having at this position large hydrophobic amino acids that do not lead to cleavage of the initiator methionine. Only leucine, phenylalanine, isoleucine, tryptophan and methionine at position 2 did not affect the mitochondrial import of our dominant negative Hsp60p-13myc allele.

Strikingly, mutagenesis of position 2 of the rat aldehyde dehydrogenase (Aldh) MTS, whose first residues are MLRAAL, yielded similar results to those we obtained by mutating Hsp60p in yeast [55]. Replacement of leucine at position 2 with a valine, which is also a hydrophobic residue but which drives the removal of iMet, did not allow efficient *in vitro* import of Aldh, whereas isoleucine, tryptophan, or phenylalanine at position 2 had no impact on mitochondrial import. Interestingly, the correct import of the L2V mutant was restored when it was translated using a system that did not remove the hydrophobic iMet. Similarly, a mutation that increased the N-terminal hydrophobic surface area compensated for the L2V mutation. These data underscore the general importance during eukaryotic evolution of the N-terminal hydrophobicity of MTSs and the necessary balance with positive charges that are also critical for precursor translocation into mitochondria. In this context, it would be crucial to further study the evolution of MTSs in order to better understand the characteristics that allow their optimized targeting to mitochondria.

### The characteristics of MTSs allow their efficient recognition by the N-terminal acetyltransferase NatC

In this work, we discovered the specific and optimal N-terminal signature of mitochondrial targeting sequences, defined by the presence of a hydrophobic amino acid directly after the uncleaved initiator methionine. This led us to hypothesize that, in *S*. *cerevisiae*, mitochondrial precursors with a MTS could be cotranslationally targeted by NatC.

Interestingly, previous analyses of the N-terminal acetylome in *S*. *cerevisiae* detected a few mitochondrial precursors, most of which had a fully acetylated ML- N-terminus [12,56]. However, even the most recent global analysis of acetylation losses upon NatC depletion did not generalize this observation, as only 8 mitochondrial precursors were ultimately detected as substrates for NatC [12]. Therefore, we used the Selective Affinity Purification of Translating Ribosomes (sel-TRAP) technology to identify nascent chains co-immunoprecipitated with NatC. In this experiment, 30% of the 402 captured nascent chains identified by their corresponding mRNA were mitochondrial precursors with a MTS, most of them having a leucine or phenylalanine at position 2. However, the nature of the residue at position 2 may not be the sole determinant of the affinity of NatC for these precursors.

The structure of NatC bound to a *S*. *cerevisiae* ligand highlighted the importance of residue at position 3 of the substrates that is fully involved in the binding in the catalytic pocket [57]. More specifically, the analysis of the ligand bound structure revealed that when an arginine is present at position 3 of the peptide, it binds to a specific site in the catalytic pocket and strongly increases the catalytic efficiency of NatC towards the peptide measured in a catalytic assay. Hence, many mitochondrial precursors have features for optimal NatC binding (retention of iMet, hydrophobic residue at position 2 and arginine at position 3), as depicted in Fig 6. As a matter of fact, one-third of MTSs that have a leucine at position 2 also have an arginine at position 3.

In summary, it appears that many MTSs possess N-terminal features that are optimal for efficient binding and acetylation by NatC. Using sel-TRAP technology, we have indeed demonstrated that MTSs are recognized co-translationally by NatC, suggesting their early N-terminal acetylation. More surprisingly, we also observed that some MTSs without features compatible with binding to the active site of NatC, i.e., those for which the i-Met is cleaved, were enriched in our sel-TRAP data. It is unlikely that these MTSs are non-canonical substrates of NatC and interact with its active site. It is then tempting to speculate that other features common to all MTSs, for example their amphipathic nature, might be involved in their interaction with NatC via alternative binding sites.

### A role for NatC in mitochondrial biogenesis?

From yeast to human, phenotypic analyses of cells lacking NatC have highlighted its critical importance for mitochondrial function. In *S*. *cerevisiae*, deletion of any of the NatC subunits has been shown to induce growth defects on non-fermentable carbon sources, such as glycerol and ethanol [57–61]. Our observation that these growth defects are significantly increased when ATP synthase is inhibited by oligomycin further emphasized the strong dependence of yeast on NatC for energy supply from mitochondrial metabolism (S4A Fig).

The importance of NatC for mitochondrial function was also underscored by the observation that depletion of NatC catalytic subunit, Naa30, in a human cell line resulted in reduced levels of mature mitochondrial matrix membrane proteins, many of whose precursors were substrates of NatC [11]. This phenotype was associated with loss of mitochondrial membrane potential and fragmentation of mitochondria.

Furthermore, in glioblastoma-initiating cells (GICs), that drive tumorigenesis of the most common primary brain malignancy, a strong overexpression of the protein Naa30 was detected [62]. shRNA-mediated knockdown of *NAA30* gene in GICs demonstrated that NatC may serve as a therapeutic target in these types of cancer, as intracranial transplantation of such NatC depleted cells resulted in prolonged animal survival compared to control mice transplanted with unmodified GICs [62]. At the cellular level *NAA30* knockdown in GICs cells results in reduced viability and decreased mitochondrial hypoxia tolerance, with a more abrupt and severe mitochondrial membrane depolarization compared to control cells in such condition. These observations suggested that NatC contribute to the stability of the mitochondrial potential, which was supported by the observation that several genes involved in hypoxia response, including HIF1α, a central regulator of transcriptional response to hypoxia, were upregulated in absence of NatC even in normal culture conditions.

At the molecular level, N-terminal acetylation by NatC was shown to modulate proteins ability to interact with other proteins or membranes allowing protein complexes formation or targeting to specific subcellular subsites. Examples of such NatC-dependent interactions include the formation of the Gag capsid of the yeast L-A helper virus [31,63], or of the Ubc12-Dcn1 complex [64,65], the insertion of the tRNA-specific methyltransferase Trm1-II into the inner nuclear membrane [66], the targeting of the human ADP-ribosylation factor-like protein 8B to lysosomes [67], or the localization of Arf-related protein 1 (hARFRP1) and its yeast orthologue yArl3 to the trans-Golgi via an interaction of their acetylated N-terminus with the hSys1/Sys1p protein [68,69].Furthermore, N-terminal acetylation of proteins was shown to function as a signal in the N-end rule pathway, which related the *in vivo* half-life of a protein to the identity of its N-terminal residue [15,19–22]. In particular, it has been shown that NatC acetylation of the initiator methionine, when followed by large hydrophobic amino acids, targets proteins to the ubiquitin-proteasome system [19] and is responsible for the observed short half-lives of reporter proteins with such N termini [70].

We have shown that, in yeast *S*. *cerevisiae*, a large proportion of MTS-bearing proteins are susceptible to acetylation by NatC during translation. However, although the substitution of leucine at position 2 has a critical impact on the import of Hsp60p into mitochondria, we observed that depletion of NatC had only a minor effect on the accumulation of the Hsp60p precursor. This result indicates that, at least for Hsp60p, the hydrophobic nature of the residue at position 2 of the MTS is more physiologically significant than its N-terminal acetylation by NatC. However, it should be noted that HSP60 mRNA was poorly enriched in our NatC Sel-TRAP experiments and therefore Hsp60 may not be the best model protein to analyze the impact of NatC on mitochondrial proteins import. More systematic analyses of mitochondrial import defects in the NatC mutant will be required to properly address this question.

A previous study of the GFP localization pattern of potential NatC substrates, including three mitochondrial proteins, failed to identify altered localization in yeast lacking the catalytic subunit of NatC [56]. However, the fluorescent imaging approach used in this study may not be sensitive enough to detect subtle mitochondrial import defects. Given the mitochondrial phenotypes upon NatC depletion, we propose that N-terminal acetylation of MTSs may optimize their ability to interact with the Tom20p receptor by increasing their N-terminal hydrophobicity. It is also possible that N-terminal acetylation has more or less effect on the efficiency of mitochondrial addressing, depending on other characteristics of the protein to be imported, including the overall N-terminal hydrophobicity of its MTS. Another non-exclusive hypothesis is that N-terminal acetylation by NatC of mitochondrial precursors may be involved in limiting their accumulation in the cytoplasm by controlling their half-life via the N-end rule proteasomal pathway. Finally, the mitochondrial phenotypes of NatC depleted cells may also be caused by degradation or dysfunctionality of one or more NatC substrates independently of their mitochondrial targeting. A systematic exploration of the impact of NatC depletion on the various MTS-bearing precursors is still needed to link the strong mitochondrial phenotypes of NatC-depleted cells to the specific role of N-terminal MTS acetylation on mitochondrial precursors fate.

## Material and methods

### *S*. *cerevisiae* strains, plasmids, and growth conditions

All *Saccharomyces cerevisiae* strains used in this study are described in Supplementary Methods (Table A in S1 Supplementary Methods). The plasmids used for yeast transformation are also described in the Supplementary Methods (Table B in S1 Supplementary Methods) as well as the oligonucleotides used for the diverse genetic constructs (Table C and D in S1 Supplementary Methods).

The strains used for the sel-TRAP experiment were derived from BY4741 by homologous recombination with the ProtA-His5 cassette amplified from the pBXA plasmid [71]. The temperature-sensitive and respiratory-deficient strain YPH499 pam16Δ-MAGN76D was kindly provided by Agnes Delahodde with the corresponding reference strain YPH499 [40]. The substitutions of the leucine at position 2 of Hsp60p were obtained by site-directed mutagenesis using CRISPR/CAS9 technology. YPH499 strains were co-transformed with the plasmid pAEF-HSP60 [72] encoding the Cas9 endonuclease and expressing a guide RNA targeting the HSP60 sequence, and a HSP60 repair template including the desired mutation. The resulting strains were used for PCR amplification of the mutant HSP60 sequences to obtain the diverse pHSPmyc(X) plasmids (X representing the amino acid at position 2 of Hsp60p sequence) by cloning into pZMYA7 [36], a pRS416-derived plasmid allowing C-terminal tagging with the 13Myc epitope.

Unless otherwise indicated, yeasts were grown at 30°C on rich medium (1% bactopeptone, 1% yeast extract) with 2% glucose. When mentioned, glycerol was used as an alternative carbon source and oligomycin (0.25μg/ml) was added. When required for plasmid selection, cells were grown on CSM medium (0.17% yeast nitrogen base, 0.5% ammonium sulfate, 2% dextrose, 0.07% CSM mixture) depleted of the selective compound. *Hygromycin* B (200 μg/ml) was used in rich medium to select mutants containing pAEF5-derived plasmid after CRISPR/CAS9 procedure. After genome sequencing verification, plasmid was removed from HSP60 mutant strains by overnight culture in rich medium without antibiotic selection.

### Antibodies

The following antibodies were used for western blotting or immunoprecipitation: rabbit IgG-HRP polyclonal antibody (PAP; code Z0113; Dako), rabbit anti-Hsp60p and mousse anti-Atp2p polyclonal antibody (gift from J.P. Di Rago), anti-Pgk1p mouse monoclonal antibody (ab113687, abcam) and mousse moclonal anti-myc antibody (11667149001, Roche).

### Data for the *in-silico* analyses

The 6,050 sequences of *S*. *cerevisiae* proteome (proteome ID: UP000002311) were downloaded from the UniProtKB database [73] (http://www.uniprot.org/uniprot/?query=proteome:up000002311&format=fasta). Since proteins encoded by transposons have redundant sequences and their distribution vary depending on yeast strains [74], the 91 proteins annotated as transposons according to the Saccharomyces Genome Database (https://www.yeastgenome.org/) were removed from the proteome.

Gene Ontology annotations were downloaded from the GO consortium database [75,76] (http://current.geneontology.org/annotations/sgd.gaf.gz). For extensive analysis of mitochondrial proteins, the 1,190 proteins belonging to the mitochondrion Gene Ontology term (GO:0005739) were manually curated: proteins that were associated to mitochondria based on only one high-throughput experiment were removed, leaving 726 proteins for which association with mitochondria was strongly evidenced. Curated mitochondrial proteins were then sorted depending on whether they carry an N-terminal mitochondrial targeting sequence, according to sequence annotations provided by the UniProtKB database [73]. 361 over 726 mitochondrial proteins were annotated as carrying a MTS (S2 Table).

For the comparative analysis, 17 species were selected from the *Saccharomycotina* yeast lineage [26] (see Table E in S1 Supplementary Methods for the list of selected species). Ortholog groups recently established for 322 budding yeast species [27] were used to retrieve the 311 groups containing a mitochondrial protein from *S*. *cerevisiae*, among which 161 had an N-terminal MTS. More than 95% of these groups (298) contained orthologs for at least 13 of the 17 selected species, and all recovered orthologs for at least 9 of these species.

### HGT scores derived from hypergeometric test

Hypergeometric tests (HGT) have been classically used to identify significant differences between the observed frequencies (F_obs_) in various subsets of interest and the reference frequency (F_ref_) of the corresponding input set. The p-values provided by these tests were then used to calculate hypergeometric scores (HGT scores) as follows: |HGT score| = |log_10_(p-value)|, with a negative sign assigned to the score in case of underrepresentation (F_obs_< F_ref_).

### Analysis of amino acids usage bias at position 2 in the proteome

For each of the 20 amino acids, the frequency of use at position 2 (F_2_) was calculated and compared with the respective average use of the amino acid in the proteome (F_mean_). Differences were statistically evaluated using hypergeometric tests and HGT scores were calculated. The observed frequency value at position 2 was also used to define a reference value called F_2ref_ calculated as follows: if F_2_>F_mean_, F_2ref_ = 0.9*F_2_, otherwise F_2ref_ = 1.1*F_2_. The F_2ref_ value was then used to test the observed frequencies at any other position and calculate an aspecificity score, representing the percentage of positions with a frequency greater than F_2ref_ when F_2_>F_ref_, or less than or equal to F_2ref_ when F_2_≤F_ref_. To calculate this score, the first 500 positions of the proteome were analyzed by a 100-position long sliding window. At each step of the analysis, the window slides by one position and the number of positions within the window with a frequency value higher (when F_2_>F_ref_) or lower (when F_2_≤F_ref_) than the reference value F_2ref_ was evaluated, to calculate a local aspecificity score. The final retained aspecificity score corresponds to the maximum value of the measured local aspecificity scores.

### Analysis of the amino acid usage bias at position 2 in subsets of proteins of the Gene Ontology

We have implemented a set of functions in R to detect gene ontology terms showing, among their associated proteins, a significant and specific amino acid usage bias at position 2. The gene annotations were downloaded from the GO consortium and stored in MGSA objects (R package MGSA [77]). The second residue associated with each protein was extracted from the downloaded proteome (UniProtKB database). For each GO domain (cellular component, molecular function, biological process), contingency tables were generated that count, for all GO terms, the number of proteins with a given amino acid at position 2. To avoid detecting amino acid usage bias due to proteins with the same N-terminal sequence, proteins sharing the same first 10 residues were grouped under the same identifier, which was counted only once. These contingency tables were used to calculate HGT scores and detect GO terms with significant over-representation relative to the proteome (HGT score>4) of one or more amino acids in position 2 of their associated proteins. This initial GO term list was reduced in several steps (see Figs 1B1 and S2). GO terms that showed the lower over-representation (fold change <1.8) and mainly correspond to GO term regrouping very large number of proteins were first removed from this initial list. Then, to reduce the redundancy of the detected GO terms, we developed a two-step algorithm to (1) select the minimum number of low-redundant BestN GO terms (overlap of GO terms with larger ones <40%) maximizing the number of proteins associated with the detected amino acid usage biases, and (2) identify smaller low-redundant BestF GO terms (overlap with larger GO term<30%) that are partially or fully included in one BestN GO terms (overlap≥40%) but have at least 30% higher frequency bias. Finally, to verify that the amino acid usage bias detected in a given GO term was not due to a specific N-terminal composition of the associated proteins, the search for position-specific amino acid usage bias was extended by calculating the HGT enrichment scores relative to the proteome on the first 100 positions of these proteins. Details of the GO reduction algorithm are available in S1 Supplementary Methods.

### Selective Translating Ribosome Affinity purification (sel-TRAP)

For analysis of canonical ribosomes and NatA, NatB and NatC-associated ribosomes, ribonucleoparticle purifications from cells expressing proteinA-tagged baits (respectively Rpl16Ap, Ard1p, Nat3p and Mak3p) were performed essentially as described in [36]. Briefly, frozen grindates obtained from cells grown in rich medium were homogenized in nine volumes of extraction buffer (20 mM Hepes pH 7.5, 110 mM KOAc, 2 mM MgCl_2_, 0.1% Tween-20, 0,5% Triton X-100, 1 mM DTT, 1× protease inhibitors cocktail, complete EDTA-free, Roche and antifoam B, Sigma, 1:5000). The obtained extract was clarified by filtration through 1.6 μm GD/X Glass Microfiber syringe filters (25 mm, Whatman) and input samples were collected for analysis of total cellular RNA and protein content. The immunoprecipitation of proteinA-tagged baits was performed by further incubation of the extract for 30 min at 4°C with IgG-conjugated magnetic beads. Beads were then washed three times with extraction buffer, once with washing buffer (0.1 M NH4OAc, 0.1 mM MgCl_2_) supplemented with 0.02% Tween-20 and four times with washing buffer without Tween-20. Beads were then split into two samples for protein and RNA analysis respectively. Immunoprecipitated proteins were eluted with 0.5 M NH_4_OH, 0.5 mM EDTA, lyophilized and resuspended either in SDS-sample buffer for SDS-PAGE or in 25 mM ammonium carbonate for mass spectrometry analysis. Immunoprecipitated RNA was eluted by using the RLT buffer from Quiagen RNeasy Mini Kit@ that was used to purified total and immunoprecipitated RNAs.

### Mass spectrometry analysis of sel-TRAP experiment

Mass spectrometry analysis procedures, performed at Proteomics Core Facility of Institut Jacques Monod, are described in S1 Supplementary Methods, and mass spectrometry proteomic data were deposited on the ProteomeXchange Consortium [78] via the PRIDE partner repository with dataset identifier PXD034922.

Mascot score distributions of immunoprecipitations from control (unlabeled BY4741) or protein A-labeled strains were compared to distinguish specifically interacting proteins from background. Based on the distribution of Mascot scores in the control, a cut-off score of 250 was chosen to retain less than 5% false positives. Only proteins with a Mascot score greater than 250 in the immunoprecipitation data of protein A-labeled strains were considered for further analysis. Proteins with a score greater than 250 in the control or with a ratio of protein A-labeled strain to control less than 2 were excluded in the final list of partners detected in sel-TRAP experiments.

Venn diagrams obtained from two independent experimental sets were used to compare the protein partners of Ard1p, Nat3p and Mak3p and determine the shared and specific partners observed in a reproducible manner. In this representation, for a more accurate definition of partners, all proteins with at least a 5-fold higher Mascot score in one of the sel-TRAP experiments compared with the others was assigned as specific partner of the corresponding immunoprecipitated protein.

### Microrray analysis of sel-TRAP experiment

The microarray data and the related protocols are available at ArrayExpress website (https://www.ebi.ac.uk/arrayexpress/experiments/E-MTAB-11772/) with the dataset identifiers E-MTAB-11772.

After sel-TRAP experiment, input and immunoprecipitated (IP) RNAs were purified using the RNeasy Mini Kit (Qiagen). One μg of RNA from each sample was then reverse-transcribed and labeled with Cy3 or Cy5 dye using the indirect labeling procedure. cDNAs of input and IP samples were then hybridized on Agilent’s ab 8×60K *S*. *cerevisiae* custom DNA chip (AMADID: 027945). Arrays were read using an Agilent scanner at 2 μm resolution and the signal segmentation was done using the feature extraction software (Agilent). The data was normalized without background subtraction using the global Lowess method [79].

To access the specifically enriched mRNAs in the Ard1p, Nat3p, or Mak3p sel-TRAP experiments, the measured log2(IP/input) ratios were corrected by subtracting the corresponding value obtained from the Rpl16Ap immunoprecipitation. Average specific enrichment values were then calculated from two independent experiments. The obtained data were filtered according to the mRNA enrichment values using a threshold value increasing in 0.1 steps from -3 to +4. From the lists of mRNAs detected above these thresholds, the evolution of the proportions of the 20 amino acids at position 2 of the corresponding proteins was analyzed to identify any possible enrichment related to this position. HGT scores were also calculated for each threshold value to validate the statistical significance of the detected enrichments. After this analysis, a maxima of the HGT scores were observed for an enrichment threshold value around 0.8 (see Fig 4A) and this value was then used for subsequent analyses of Mak3-associated mRNAs. The LIMMA algorithm [80,81] statistically validated the reproducibility of the list of enriched mRNA obtained with this threshold: all mRNAs with a mean log2 ratio greater than 0.8 in the two independent sel-TRAP experiments had an adjusted p-value lower than 0.05.

### Crude protein extract and cell fractionation

Crude protein extracts were obtained by the conventional TCA extraction method. Cell lysis (3 OD_600_ grown overnight in glucose medium) and protein precipitation were performed by two successive incubations of 10 minutes on ice in 0.24 M NaOH, followed by the addition of 5% TCA. Cell debris was removed by centrifugation and the protein pellet was directly resuspended in a fresh mixture of 60 μL 2X Laemli, 15 μL 1M Tris Base, and 2% β-mercaptoethanol.

Cell fractionation method was adapted from classic mitochondrial extraction protocol [82]. Spheroplasts were obtained from cells grown in glucose medium and incubated in digestion buffer (1.2M Sorbitol, 50mM Tris-HCl pH7.5, 10mM EDTA, 0.3% β-mercaptoethanol) with 1 unit of lyticase (Sigma-Aldrich) per OD_600_ for 1 hour at 30°C. After centrifugation (5000rpm, 15 min, 4°C), cells were resuspended in 20ml extraction buffer (0.7M Sorbitol, 50mM Tris-HCl pH7.5, 0.2mM EDTA, 1× complete EDTA-free Protease Inhibitor from Roche) by 10 up and down pipetting cycles. The total input sample was obtained by mixing 20μl of spheroplast extracts with 20μl of 2X Laemmli buffer. Cell remnants and nucleus were removed by low-speed centrifugation (3500rpm, 5 min, 4°C) and mitochondrial and cytosolic fractions were separated by high-speed centrifugation (11,000 rpm, 30 min, 4°C). Mitochondria were washed once before finally being resuspended in 100μl of extraction buffer.

## Supporting information

S1 Table Complete table of GO terms identified with amino acids biases at position 2. (XLSX)

S2 Table Annotation of mitochondrial proteins of *S*. *cerevisiae*. (XLSX)

S3 Table Microarray data from sel-TRAP experiments. (XLSX)

S4 Table Mass spectrometry data from sel-TRAP experiments. (XLSX)

S5 Table Western blot quantification of Hsp60p precursor/mature ratio. (XLSX)

S1 Supplementary Methods The S1 Supplementary Methods pdf file contains the following tables: Table A: Yeast strains. Table B: Plasmids and derived strains. Table C: Oligonucleotides. Table D: Codon used in the reparation cassette to encode the desired X mutation. Table E: List of the yeast species used for the genomic comparative studies
(PDF)

S1 Fig Amino acid usage preferences at position 2 are conserved in the *Saccharomycotina* lineage. (A) Heatmaps of HGT scores showing conservation of preferences for amino acids at position 2 in 17 budding yeasts of the *Saccharomycotina* lineage. In each proteome, HGT scores were calculated to compare amino acid usage at position 2 to their respective average usage at any position (A1). HGT scores were also calculated for positions 3 to 30 to extract the maximum HGT score and confirm the specificity of preferences for amino acids at position 2 (A2). (B) Heatmaps of HGT scores showing that preferences for large hydrophobic amino acids are restricted to position 2 in the MTSs of 17 budding yeasts of the *Saccharomycotina* lineage. In each species, the maximum HGT scores for positions 3 to 30 were calculated to identify amino acid usage preferences relative to amino acid usage in the proteome at these same positions. In contrast to the analysis of amino acid preferences at position 2 (Fig 2C), no enrichment for Leu, Phe, or Ile was detected at positions 3 to 30.
(TIF)

S2 Fig Schematic representation of the GO term selection procedure. (A) GO term selection procedure illustrated focusing on one particular amino acid *a* (in green). GO terms are first extracted on the basis of their significant overrepresentation of amino acid a at position 2 assessed by hypergeometric test (HGT score > 4). The initial list of enriched GO term is reduced by applying several filters: (1) elimination of GO terms with the lowest frequency bias (<1.8 fold change), (2) selection of BestN GO terms encompassing the largest number of proteins and maximizing the coverage of the original dataset (Steps 1 and 2 in B panel), (3) complementation of bestN GO terms with one or more smaller BestFGO terms displaying the highest position 2 frequency biases terms (Steps 3 and 4 in B panel). (B) Detailed procedure of BestN and BestF GO terms selection. Each circle represents the set of proteins associated with a given GO term. The colored patches indicate the proportions of proteins displaying specific amino acids in position 2. Each color stands for an amino acid (amino acid a in green, b in yellow and c in blue). At each step, the GO terms are ordered according to their scores, which reflect coverage and/or selectivity. Some GO terms are eliminated (red crosses) because their protein sets substantially overlap with the previous one(s). In step 2, new GO terms coming from the analysis of other amino acids can be added (red circle). In step 4, any GO term identified in the BestF list may be replaced by a GO term representing better a set of amino acids (orange circle). See more details in S1 Supplementary Methods.
(TIF)

S3 Fig Specific N-terminal signature detected in GO terms corresponding to cellular pathways and molecular function. Bar graph showing the percentages of use of the 20 amino acids at position 2 in GO terms corresponding to cellular pathways (A) and molecular functions (B) showing preferences at position 2 for specific amino acids. All BestN (bold type) and BestF (normal type) GO terms selected by our algorithm are shown. Significant overrepresentations of amino acids (HGT score > 3) are highlighted. Amino acids are sorted in decreasing order of use at position 2 in the proteome.
(TIF)

S4 Fig Respiratory phenotypes of *mak3Δ* and *mak3-PA* strains. (A) The growth defect of the *mak3Δ* strain on glycerol medium is significantly enhanced by the inhibition of mitochondrial ATP synthase by oligomycin. (B) C-terminal PA Tag required for the sel-TRAP experiment did not disrupt cell growth on glycerol medium. Strains were grown on Glucose medium and diluted in water to 0.5 (A) or 0.2 (B) OD_600_. Tenfold serial dilutions were plotted (5μl) on glucose or glycerol medium optionally supplemented with oligomycin (0.25μg/μl). Temperature and incubation time are indicated. The *mak3Δ* and mak3-PA strains were derived respectively from the parental strains YPH499 and BY4741 (see Table A in S1 Supplementary Methods).
(TIF)

S5 Fig Analysis of the selectivity of NatA, NatB and NatC toward proteins N-termini using the sel-TRAP technology. Analysis of the proportion of N-termini, defined as the amino acid observed at position 2, in proteins encoded in the transcriptomic data from Ard1-PA (A), Nat3-PA (B) and Mak3-PA (C) immunoprecipitation confirmed that sel-TRAP experiments have specifically purified canonical substrates of NatA, NatB and NatC respectively. Threshold-independent analyses showing for each amino acid the proportion of corresponding N-termini as a function of enrichment value in the sel-TRAP data are shown in A1, B1, and C1. For each x-axis value, the y-value shown is the % of N-termini among those encoded by mRNAs with an enrichment value greater than or equal to the x value. An enrichment value threshold of 0.8 was used to analyze a set of putative targets of each Nat (A2, B2, C2). The distribution of the different N-termini among putative targets (upper panel in A2, B2 and C2) showed enrichments for residues defining canonical substrates of each Nats (i.e. S at position 2 for NatA, D, N, E and Q at position 2 for NatB, and F, L, I and W at position 2 for NatC). Statistical evaluation of the observed enrichment was performed by calculating HGT scores (lower panel in A2, B2 and C2) and confirmed a significant enrichment at position 2 (HGT≥3, dotted line) in serine (S) for NatA, aspartate (D) for NatB, and phenylalanine (F) and leucine (L) for NatC.
(TIF)

S6 Fig Quantification procedure of Hsp60p precursor in Western Blot images. (A) The quantification procedure is illustrated on the western blot shown in Fig 5A1. We developed a basic ImageJ macro to collect in each sample area the average intensity values measured in a sliding window from 30 pixels upstream to 30 pixels downstream of the band corresponding to mature Hsp60p. (B) For each sample, the distribution of the average intensity value was plotted along the x-axis, allowing determination of the peak intensity for the precursor (P) and mature (M) bands. Because the precursor and mature intensity Gaussians overlap, we systematically corrected the precursor intensity value by the symmetric value on the x-axis to avoid overestimating the precursor band accumulation. (C) This accumulation was estimated by comparing the ratio of precursor to mature Hsp60p signal in the mutant strains compared to the reference WT strain.
(TIF)

S7 Fig Mitochondrial precursors that accumulate in L2D mutant are localized within the cytoplasm. Cellular fractionations from three independent cultures were performed from the L2D mutant strain and its parental YPH499 pam16Δ-MAGN76D strain. The L2D mutant was chosen for these experiments because it presented the highest rate of accumulation of the precursor (closed to 80% of the total Hsp60 signal according Fig 5A2). Input protein samples were obtained by directly mixing spheroplasts after cell wall digestion with 2X Laemmli buffer. For comparison, crude TCA extracts were also obtained from the same culture before digestion. Cytosolic and mitochondrial protein samples were obtained after differential centrifugations by mixing the corresponding fractions with 2X Laemmli buffer. (A) Western blot images obtained after hybridization of antibodies directed against Hsp160p, Atp2p, and Pgk1p and simultaneous acquisition of the signals from the different antibodies. The downward and upward pointing arrows indicate, in each lane, the precursor and mature bands of Hsp60p respectively. Note that the TCA extraction method caused a shift in protein migration compared with the fractionation samples. Mature Hsp60p observed in WT strain is highly accumulated in mitochondrial fractions, whereas Pgk1p protein is mainly detected in the cytosolic fraction in both strain samples. Hsp60p precursor that highly accumulated in L2D mutant is only detected in the cytosolic fraction, confirming that it is not imported into mitochondria. (B) Western blot images obtained after independent antibody hybridizations. Before incubation with antibodies, the membrane was cut into three parts to separate the areas corresponding to the localization of Hsp160p, Atp2p and Pgk1p. This allowed us to improve the signal for each protein by optimizing the image acquisition time and to confirm the localization of the Hsp60p precursor in the cytosol.
(TIF)

S8 Fig Western blot analysis of protein content in clones obtained after transformation with the diverse pHSPmyc(X) plasmids. Protein extracts were analyzed by SDS-PAGE followed by western blots using antibodies against myc epitope, Hsp60p, and Pgk1p. The position of the precursor and mature Hsp60p are indicated. (A) Analysis of clones transformed with plasmid expressing a toxic Hsp60p-13myc protein (X = L, F, I, M, W, see Fig 5B) and of those obtained with the pHSP60(Y) for which a longer exposure time was necessary to detect the Hsp60p-13myc protein (see S8B Fig). (B) Analysis of clones transformed with plasmids that allow the rescue of the dominant negative toxicity of the Hsp60p-13myc protein (X = D, E, N, Q, H, K, R, Y, S, A, T, C, P, G, V, see Fig 5B).
(TIF)
