# Supplementary material for: Functional mapping of N-terminal residues in the yeast proteome uncovers novel determinants for mitochondrial protein import
Source: PLoS Genet. 2023 Aug 16;19(8):e1010848. doi: 10.1371/journal.pgen.1010848 (PMC10482271; doi:10.1371/journal.pgen.1010848)
Supplement: S1 Supplementary Methods — The S1 Supplementary Methods pdf file contains the following tables: Table A: Yeast strains. Table B: Plasmids and derived strains. Table C: Oligonucleotides. Table D: Codon used in the reparation cassette to encode the desired X mutation. Table E: List of the yeast species used for the genomic comparative studies (PDF) [file pgen.1010848.s006.pdf]

Functional mapping of N-terminal residues in the yeast proteome uncovers novel determinants for mitochondrial protein import

Salomé Nashed[1*], Houssam El Barbry[1*], Médine Benchouaia[1], Angélie Dijoux-Maréchal[1], Thierry Delaveau[1], Nadia Ruiz-Gutierrez[1], Lucie Gaulier[1], Déborah Tribouillard-Tanvier[3], Guillaume Chevreux[2], Stéphane Le Crom[1], Benoit Palancade[2], Frédéric Devaux[1], Elodie Laine[1], Mathilde Garcia[1**]

[1] Sorbonne Université, CNRS, Institut de Biologie Paris-Seine, UMR 7238, Laboratoire de Biologie Computationnelle et Quantitative, Paris, France

[2] Université Paris Cité, CNRS, Institut Jacques Monod, F-75013 Paris, France.

[3] Université de Bordeaux, CNRS, IBGC, UMR5095, F-33000Bordeaux, France

* These authors contributed equally to this work

** Corresponding author mathilde.garcia@sorbonne-universite.fr

SUPPLEMENTARY METHODS

Table A: Yeast strains

Strain	Genotype	Origin
BY4741	MATa; his3Δ1; leu2Δ0; met15Δ0; ura3Δ0	Euroscarf (Brachmann et al,1998)
BY4741 *mak3Δ*	MATa his3Δ1 leu2Δ0 met15Δ0 ura3Δ0 MAK3::KanMX4	
Ard1-PA	MATa; his3Δ1; leu2Δ0; met15Δ0; ura3Δ0, ARD1-ProtA-His5	This Study[*1]
Nat3-PA	MATa; his3Δ1; leu2Δ0; met15Δ0; ura3Δ0, NAT3-ProtA-His5	
Mak3-PA	MATa; his3Δ1; leu2Δ0; met15Δ0; ura3Δ0, MAK3-ProtA-His5	
YPH499	MATa ura3-52 lys2-801_amber ade2-101_ochre trp1-Δ63 his3-Δ200 leu2-Δ1	Mehawej et al, PLoS Genet, 2014
YPH499 pam16Δ-MAGN76D	MATa ura3-52 lys2-801_amber ade2-101_ochre trp1-Δ63 his3-Δ200 leu2-Δ2 pma16Δ + pmagMAT N76D	
YPH499 *mak3Δ*	MATa ura3-52 lys2-801_amber ade2-101_ochre trp1-Δ63 his3-Δ200 leu2-Δ1 MAK3::KanMX4	This Study[*2]
YPH499 pam16Δ-MAGN76D *mak3Δ*	MATa ura3-52 lys2-801_amber ade2-101_ochre trp1-Δ63 his3-Δ200 leu2-Δ2 pma16Δ + pmagMAT N76D MAK3::KanMX4	

*[1]Strains tagged at their C terminus were obtained from BY4741 strain, after classical procedure of lithium acetate transformation, by homologous recombination with ProtA-His5 cassette amplified from pBXA (Rout et al., 2000 , provided by M. Rout, The Rockefeller, University, New York, NY)

*[2] Mak3 deletions were obtained by homologous recombination with the KanMX4 deletion cassette amplified from the BY4741 mak3Δ strain

Table B: Plasmids and derived strains

YPH99 or YPH499 pam16Δ-MAGN76D strain were transformed with the following plasmids.

Plasmids (Origin)	Inserted sequence	Experiment
pAEF-HSP60 (This Study [*3])	Cas9 sequence<br>HSP60 Guide Sequence	CRISPR/CAS9 mutagenesis of HSP60 genomic locus
pZymA7 (1)	ZWF1 promotor<br>ASN1(1-1717)-13myc<br>PGK1 terminator	Study of the impact of L2X mutations on the dominant negative toxicity of the Hsp60p-13myc protein
pHSPMyc(X)(This study[*4])	HSP60 Promotor<br>HSP60 L2X-13myc<br>PGK1 terminator	

[*2] The plasmid pAEF-HSP60 contains the guide RNA sequence targeting HSP60 and the sequence coding for the Cas9 endonuclease. It allows to cut the genomic sequence of HSP60 to obtain the desired mutation of the second residue of HSP60 by homologous recombination with an ad hoc repair cassette (see table S3).

[*4] X represents the amino acid that replaces leucine at position 2 of Hsp60p. This modification was first introduced into the *S. cerevisiae* genome with CRISPR/CAS9 technology using HSP60 repair cassettes that include the desired mutations (see Table S3 and S4). The mutated HSP60 sequences, including its promoter (680 bp upstream of the initiator codon), were then amplified by PCR (see oligonucleotides used in Table S3) and inserted between the SacI and PacI restriction sites into the plasmid pZMYA7 in fusion with the 13myc sequence.

Table C: Oligonucleotides

Oligonucleotide Name	Sequence 5' →3'	
HSP60 guide for	**ATC**AGTAGCGCGACTACGAACAA	CRISPR/CAS9 mutagenesis of HSP60*5
HSP60 guide rev	**AAC**TTGTTCGTAGTCGCGCTACT	
K7HSP60(X)_fw	ACATCATAAGCAAAAAAGTTTTCAAA**ATG**YYYAGATCATC**A**GTTG TTCGTAGTCGCGCTACTTTAAGGCCTTTATTGCGTCGTGCTTACT	
K7HSP60(X)_rev	AGTAAGCACGACGCAATAAAGGCCTTAAAGTAGCGCGACTACGA ACAAC**T**GATGATCTZZZCATTTTGAAAACTTTTTTGCTTATGATGT	
SacI_HSP60(-800)_for	AGCCACAT**GAGCTC**GCTCACGCAGCTTACGTTCC<br>Sac	PCR amplification of HSP60 sequence for insertion in pZMYA7
PacI_HSP60(stop)_rev	AGCCACATT**TAATTAA**CATCATACCTGGCATTCCTGGCATAC<br>Pac	

*5: The forward and reverse HSP60 guide oligonucleotides were hybridized and the resulting dsDNA was inserted in LguI site (LguI extensions underlined in sequences) of the pAEF5 plasmid (2). The resulting plasmid (pAEF HSP60) was co-transformed in yeast (YPH499 or YPH499 pam16Δ-MAGN76D strain) with the repair cassette previously obtained by hybridization of the forward and reverse oligonucleotides K7HSP60. In these oligonucleotides, the codon following the initiator methionine (underlined in the sequence) was replaced with codons encoding the desired X mutation (indicated by YYY/ZZZ in the sequence, see Table S4 for the codon chosen for each mutation). In the repair cassette, a synonymous mutation was introduced into the HSP60 sequence to eliminate the Pam site targeted by the CAS9 endonuclease with the chosen guide RNA sequence.

Table D: Codon used in the reparation cassette to encode the desired X mutation

The selected codons are not rare codons in *S. cerevisiae*.

X	Amino Acid	YYY	ZZZ
L	Leucine	TTA	TAA
N	Asparagine	AAT	ATT
K	Lysine	AAG	CTT
T	Threonine	ACT	AGT
S	Serine	TCC	GGA
R	Arginine	AGA	TCT
I	Isoleucine	ATT	AAT
M	Methionine	ATG	CAT
H	Histidine	CAT	ATG
Q	Glutamine	CAA	TTG
P	Proline	CCT	AGG
D	Aspartic Acid	GAT	ATC
E	Glutamic Acid	GAA	TTC
A	Alanine	GCT	AGC
G	Glycine	GGT	ACC
V	Valine	GTC	GAC
Y	Tyrosine	TAT	ATA
C	Cysteine	TGT	ACA
W	Tryptophan	TGG	CCA
F	Phenylalanine	TTT	AAA

Table E: List of the yeast species used for the genomic comparative studies

Saccharomyces cerevisiae
Saccharomyces paradoxus
Saccharomyces bayanus
Candida glabrata
Zygosaccharomyces rouxii
Eremothecium gossypii
Kuyveromyces lactis
Lachancea thermotolerans
Lachancea kluyveri
Debaryomyces hansenii
Scheffersomyces stipitis
Clavispora lusitaniae
Ogataea polymorpha
Komagataella pastoris
Yarrowia lipolytica
Blastobotrys adeninivorans
Geotrichum candidum

GO reduction algorithm

Definitions

Let G_i be the ensemble of genes from the protein-coding fraction $\mathcal{P}$ associated with the GO term GO_i, such that $G_i = \{g \in \mathcal{P} \mid g \sim GO_i\}$, where g is a gene and the symbol $\sim$ denotes the association between a gene and a GO term. We define G_i^a as the subset of genes from G_i whose resulting proteins display the amino acid a in position 2. We evaluate the representativity of a GO term with respect to a according to the following score,

$$s_i^a(w) = w_N \frac{|G_i^a|}{\max_j(|G_j^a|)} + w_F \frac{f_i^a}{\max_j(f_j^a)}, \quad (1)$$

where $f_i^a = \frac{|G_i^a|}{|G_i|}$ is the frequency of occurrence of a in the second position of the proteins resulting from the genes included in G_i, and w_N and w_F are weights controlling the relative contributions of the two terms such that $w_N + w_F = 1$. The first term reflects how well the GO term GO_i covers the proteins displaying a in position 2. The GO term associated with the maximum number of such proteins has the maximum value of 1. The second term reflects how specific the GO annotation is for the amino acid a. The lower the variability of amino acids in position 2 among the proteins associated with a given GO term, the higher this value.

Initialization: Pre-selection of GO terms

We start from 20 sets of pre-selected GO terms corresponding to the 20 amino acids. For each amino acid a, the N_a selected GO terms are those displaying an enrichment for a with a p-value $p - val \leq 10^{-4}$, and also for which $f_i^a > 1.8 f^a$, where f^a is the frequency of occurrence of a at position 2 in the proteome. Notice that some sets may be empty.

First step: identifying the most inclusive and selective GO terms for each amino acid

The goal of this first step is to define, for each amino acid, a minimal set of GO terms representative of the full pre-selected set, both in terms of coverage and of specificity. We first order the GO terms according to the score expressed in Eq. 1, from the highest to the lowest score. We set the weight $w_N = w_F = 0.5$ so that coverage and specificity contribute equally to the score. Then, we apply the following Algorithm 1, independently for each amino acid a. The algorithm considers the N_a GO terms pre-selected for a, from the highest-scored one to the lowest-scored one, and decides whether each term should be retained or discarded based on its gene overlap with the previously retained terms. At each iteration of the algorithm, we require that the GO term considered shares less than $\alpha = 40\%$ of its associated genes with the current GO term list. If this criterion is not met, then the GO term is disregarded.

Second step: reducing redundancy at the global level while prioritizing coverage

While the first step treats each amino acid independently from the others, this second step aims at reducing redundancies across all amino acids. More precisely, for each amino acid a, we look for the GO terms not included in its $bestNGO$ list but present in the $bestNGO$ list of at least one other amino acid b and displaying an enrichment for a with a p-value $p - val \leq 10^{-3}$. We extend the $bestNGO$ list of a with these GO terms. They are of interest because they display high enrichments for several amino acids and they potentially cover more genes than the terms selected in the previous step. To allow for them to be retained, and potentially replace GO terms with lower coverage, we again apply Algorithm 1 for each amino acid setting $w_N = 1$ and $w_F = 0$.

Third step: rescuing highly selective GO terms

This goal of this third step is to rescue the GO terms displaying a very high selectivity for a particular amino acid. Here, for each amino acid a, we start from the n_a GO terms pre-selected in the initialization phase but not retained in the $bestNGO$ list. We order them according to the score expressed in Eq. 1, from the highest to the lowest score, with the weights set to $w_N = 0$ and $w_F = 1$. The rationale is to prioritize the most selective GO terms. Then, we apply the following Algorithm 2. At each iteration, we require for a GO term to be retained that it shares less than $\beta = 30\%$ of its associated genes with each one of the GO terms in the current list.

Fourth step: reducing redundancy at the global level while prioritizing specificity

Similarly to what is done in the second step, the goal of this fourth step is to reduce redundancies across all amino acids. We consider the nF GO terms appearing in at least one of the $bestFGO$ lists defined in the previous step. For each of these GO terms, we identify the amino acids for which it displays an enrichment with a $p - val \leq 10^{-4}$. This operation leads to nF potentially overlapping subsets of amino acids. The subsets with only one member are discarded. We encode this information in a graph where the nodes are the 20 amino acids and the set of edges is defined from the subsets.

Algorithm 1 Redundancy reduction applied to a list of pre-selected GO terms

Input: an ordered list of GO terms $[GO_1, GO_2, ..., GO_{N_a}]$ and the value of α

Output: a reduced list of GO terms

$bestNGO \leftarrow [GO_1]$
$bestN \leftarrow G_1^a$
for $i \leftarrow 2$ to N_a **do**
    $overlap \leftarrow |bestN \cap G_i^a|$
    if $overlap \leq \alpha|G_i|$ **then**
        Add GO_i to $bestNGO$
        $bestN \leftarrow bestN \cup G_i^a$
    end if
end for
Output the list $bestNGO$

Algorithm 2 Rescue of GO terms from a pre-filtered list

Input: an ordered list of GO terms $[GO_1, GO_2, ..., GO_{n_a}]$ and the value of β

Output: a reduced list of GO terms

$bestFGO \leftarrow [GO_1]$
for $i \leftarrow 1$ to n_a **do**
    $c \leftarrow 0$
    for $j \leftarrow 1$ to $length(bestFGO)$ **do**
        $overlap \leftarrow |G_j^a \cap G_i^a|$ # j and i are the indices of GO terms from $bestFGO$
                        and from the input list, respectively
        if $|G_j^a| \leq |G_i^a|$ **then**
            $overlapRel \leftarrow overlap/|G_i^a|$
        else
            $overlapRel \leftarrow overlap/|G_j^a|$
        end if
        if $overlapRel \leq \beta$ **then**
            $c \leftarrow c + 1$
        end if
    end for
    if $c = length(bestFGO)$ **then**
        Add GO_i to $bestFGO$
    end if
end for
Output the list $bestFGO$

Algorithm 3 Build a graph representing the overlaps between GO terms enriched for a set of amino acids

Input: a list $[GO_1, GO_2, ..., GO_m]$, a list of amino acids $[a_1, a_2, ..., a_l]$, and the value of β
Output: a graph

Initialize the graph $\mathcal{G} = (\mathcal{V}_G, \mathcal{E}_G)$ with $\mathcal{V}_G = \{GO_1, GO_2, ..., GO_m\}$
for $i \leftarrow 1$ to $m - 1$ **do**
    for $j \leftarrow i + 1$ to m **do**
        $c \leftarrow c + 1$
        for $k \leftarrow 1$ to l **do**
            $overlap \leftarrow |G_i^{a_k} \cap G_j^{a_k}|$
            if $|G_i^{a_k}| \leq |G_j^{a_k}|$ **then**
                $overlapRel \leftarrow overlap/|G_j^{a_k}|$
            else
                $overlapRel \leftarrow overlap/|G_i^{a_k}|$
            end if
            if $overlapRel \geq \beta$ **then**
                $c \leftarrow c + 1$
            end if
        end for
        if $c = l$ **then**
            Add an edge between GO_i and GO_j
        end if
    end for
end for
Output the graph $\mathcal{G}$

More specifically, any pair of nodes is linked by an edge if the corresponding amino acids were found in the same subset at least once. We further define groups of amino acids as the connected components of the graph. Each group $\{a_1, a_2, ..., a_l\}$ is associated with a set of GO terms $\{GO_1, GO_2, ..., GO_m\}$ coming from the *bestFGO* lists. We then build a graph encoding the gene overlaps between these GO terms by applying Algorithm 3. For each connected component in the graph, we determine a representative GO term GO_{imax} as the most selective one for the amino acid group. Formally, we determine $\arg\max_i(f_i^{a_1} + f_i^{a_2} + ... + f_i^{a_l})$. The final operation consists in replacing all the GO terms appearing in the *bestFGO* lists defined for the amino acids $a_1, a_2, ..., a_l$ by their representative GO_{imax}.

Mass spectrometry analysis

Digestion was performed overnight at 37°C in the presence of 12.5 µg/ml of sequencing grade trypsin (Promega, Madison, Wi, USA). Peptides mixtures were analyzed by a Q-Exactive Plus coupled to a Nano-LC Proxeon 1000 (both from Thermo Scientific). Peptides were separated by chromatography using the following conditions: Acclaim PepMap100 C18 pre-column (2 cm, 75 µm i.d., 3 µm, 100 Å), Pepmap-RSLC Proxeon C18 column (50 cm, 75 µm i.d., 2 µm, 100 Å), 300 nl/min flow rate, a 98 min gradient from 95 % solvent A (water, 0.1 % formic acid) to 35 % solvent B (100 % acetonitrile, 0.1% formic acid) followed by column regeneration, giving a total time of 120 minutes. Precursor peptides were analyzed in the Orbitrap cell in positive mode, at a resolution of 70,000, with a mass range of *m/z* 375-1500 and an AGC target of 3.10^6. MS/MS data were acquired in the Orbitrap cell in a Top20 data-dependent mode with a dynamic exclusion of 30 seconds. Fragments were obtained by Higher-energy C-trap Dissociation (HCD) activation with a collisional energy of 27% and a quadrupole isolation window of 1.4 Da. The Orbitrap cell was set at a resolution of 17,500, *m/z* 200-2000 and an AGC target of 2.10^5. Peptides with unassigned charge states or monocharged were excluded from the MS/MS acquisition. The maximum ion accumulation times were set to 50 ms for MS acquisition and 45 ms for MS/MS acquisition.

Data were processed with Proteome Discoverer 2.2 software (Thermo Fisher scientific, San Jose, CA) coupled to an in-house Mascot search server (Matrix Science, Boston, MA; version 2.5.1). MS/MS spectra were searched against the SwissProt protein database release 2017_09 with the *Saccharomyces Cerevisiae (baker's yeast)* taxonomy and a maximum of 2 missed cleavages. Precursor and fragment mass tolerances were set to 6 ppm and 0.02 Da respectively. The following post-translational modifications were included as variable: Acetyl (Protein N-term), Oxidation (M), Phosphorylation (STY). Spectra were filtered using a 1% FDR with the percolator node.

References

1. Delaveau T, Davoine D, Jolly A, Vallot A, Rouvière JO, Gerber A, et al. Tma108, a putative M1 aminopeptidase, is a specific nascent chain-associated protein in Saccharomyces cerevisiae. Nucleic Acids Res. 14 oct 2016;44(18):8826-41.

2. Agier N, Fleiss A, Delmas S, Fischer G. A Versatile Protocol to Generate Translocations in Yeast Genomes Using CRISPR/Cas9. Methods Mol Biol. 2021;2196:181-98.
