# Supplementary figures and images for: Functional mapping of N-terminal residues in the yeast proteome uncovers novel determinants for mitochondrial protein import
Source: PLoS Genet. 2023 Aug 16;19(8):e1010848. doi: 10.1371/journal.pgen.1010848 (PMC10482271; doi:10.1371/journal.pgen.1010848)

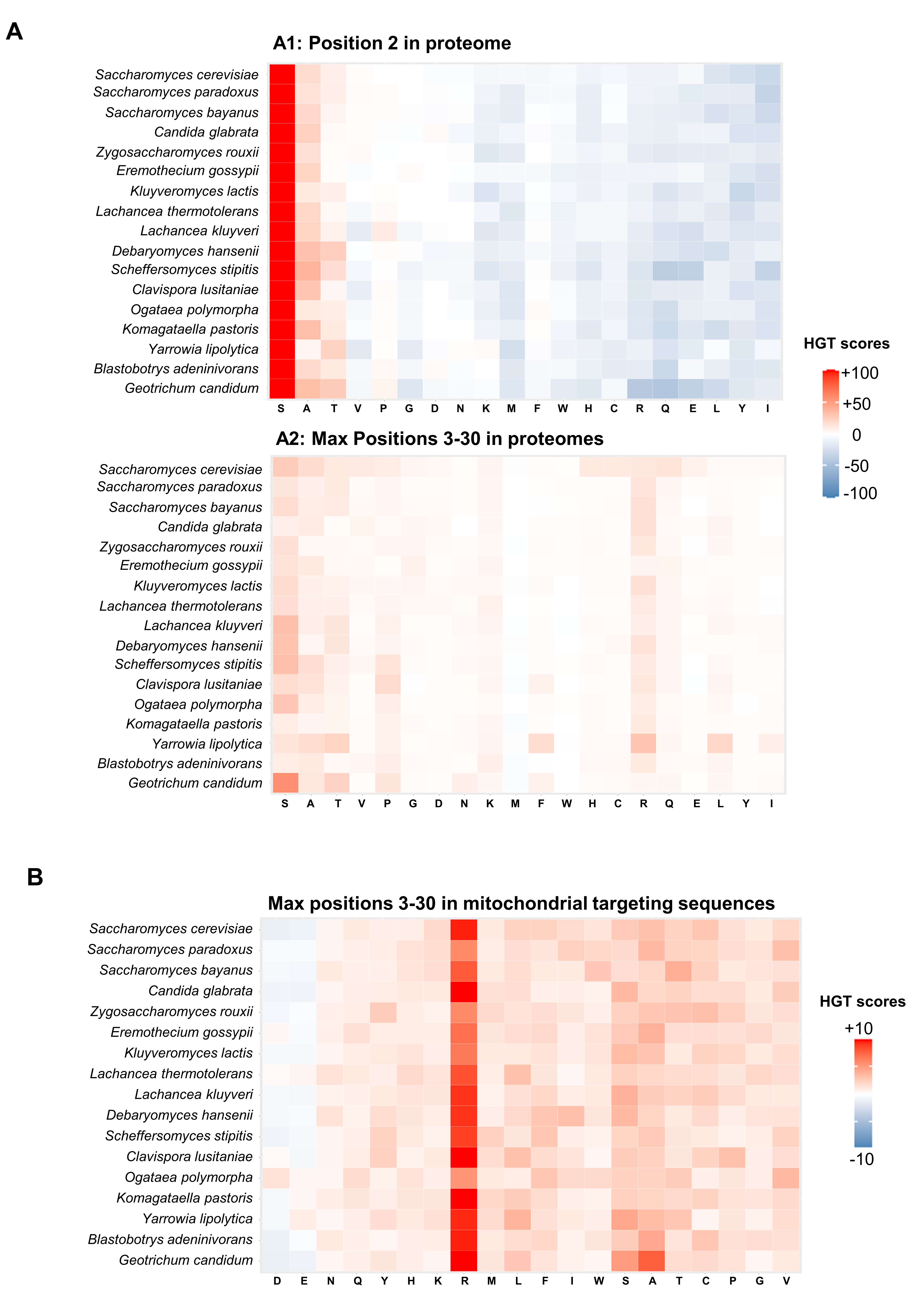

Supplement: S1 Fig — (A) Heatmaps of HGT scores showing conservation of preferences for amino acids at position 2 in 17 budding yeasts of the Saccharomycotina lineage. In each proteome, HGT scores were calculated to compare amino acid usage at position 2 to their respective average usage at any position (A1). HGT scores were also calculated for positions 3 to 30 to extract the maximum HGT score and confirm the specificity of preferences for amino acids at position 2 (A2). (B) Heatmaps of HGT scores showing that preferences for large hydrophobic amino acids are restricted to position 2 in the MTSs of 17 budding yeasts of the Saccharomycotina lineage. In each species, the maximum HGT scores for positions 3 to 30 were calculated to identify amino acid usage preferences relative to amino acid usage in the proteome at these same positions. In contrast to the analysis of amino acid preferences at position 2 (Fig 2C), no enrichment for Leu, Phe, or Ile was detected at positions 3 to 30. (TIF) [file pgen.1010848.s007.tif]

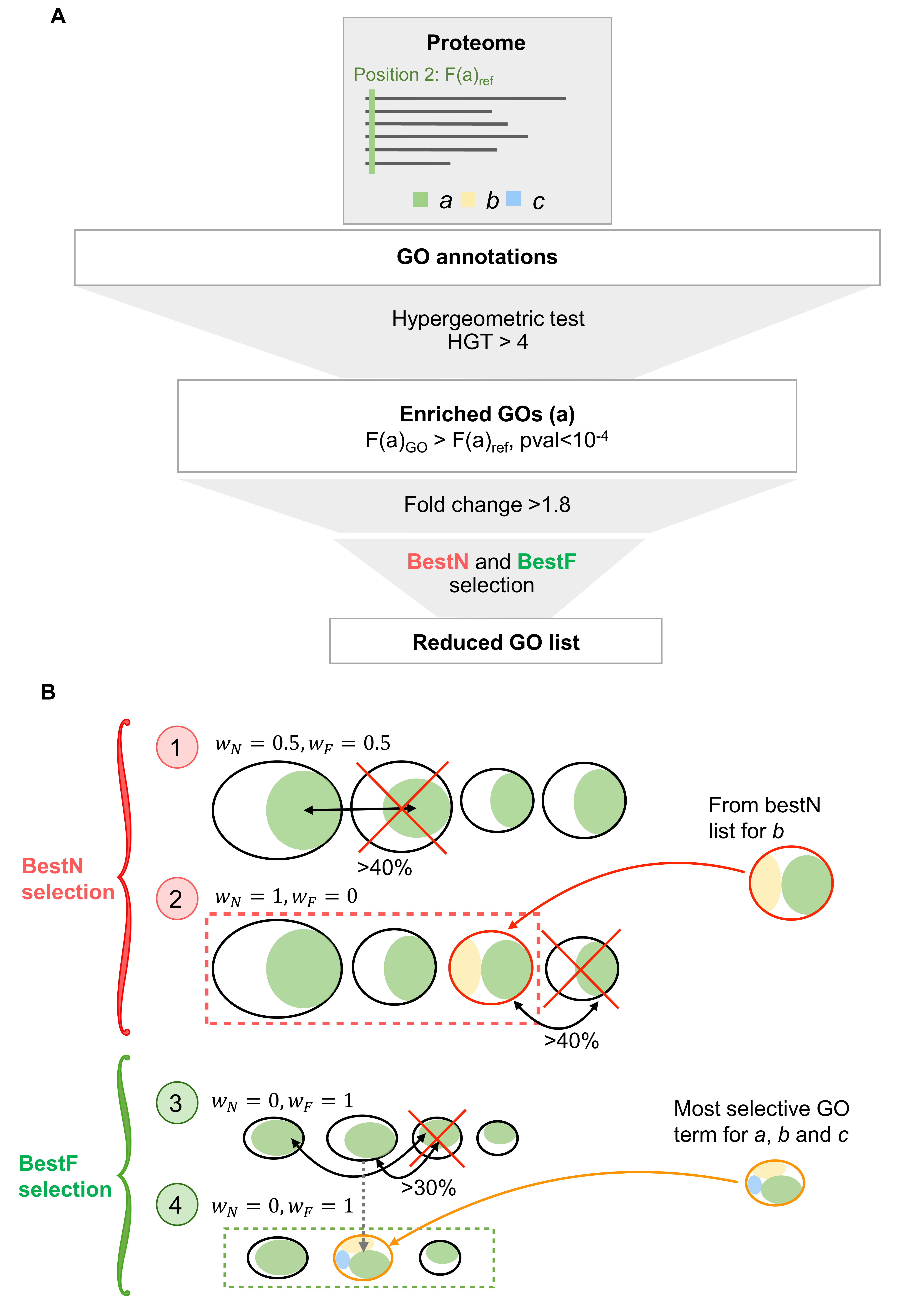

Supplement: S2 Fig — (A) GO term selection procedure illustrated focusing on one particular amino acid a (in green). GO terms are first extracted on the basis of their significant overrepresentation of amino acid a at position 2 assessed by hypergeometric test (HGT score > 4). The initial list of enriched GO term is reduced by applying several filters: (1) elimination of GO terms with the lowest frequency bias (<1.8 fold change), (2) selection of BestN GO terms encompassing the largest number of proteins and maximizing the coverage of the original dataset (Steps 1 and 2 in B panel), (3) complementation of bestN GO terms with one or more smaller BestFGO terms displaying the highest position 2 frequency biases terms (Steps 3 and 4 in B panel). (B) Detailed procedure of BestN and BestF GO terms selection. Each circle represents the set of proteins associated with a given GO term. The colored patches indicate the proportions of proteins displaying specific amino acids in position 2. Each color stands for an amino acid (amino acid a in green, b in yellow and c in blue). At each step, the GO terms are ordered according to their scores, which reflect coverage and/or selectivity. Some GO terms are eliminated (red crosses) because their protein sets substantially overlap with the previous one(s). In step 2, new GO terms coming from the analysis of other amino acids can be added (red circle). In step 4, any GO term identified in the BestF list may be replaced by a GO term representing better a set of amino acids (orange circle). See more details in S1 Supplementary Methods. (TIF) [file pgen.1010848.s008.tif]

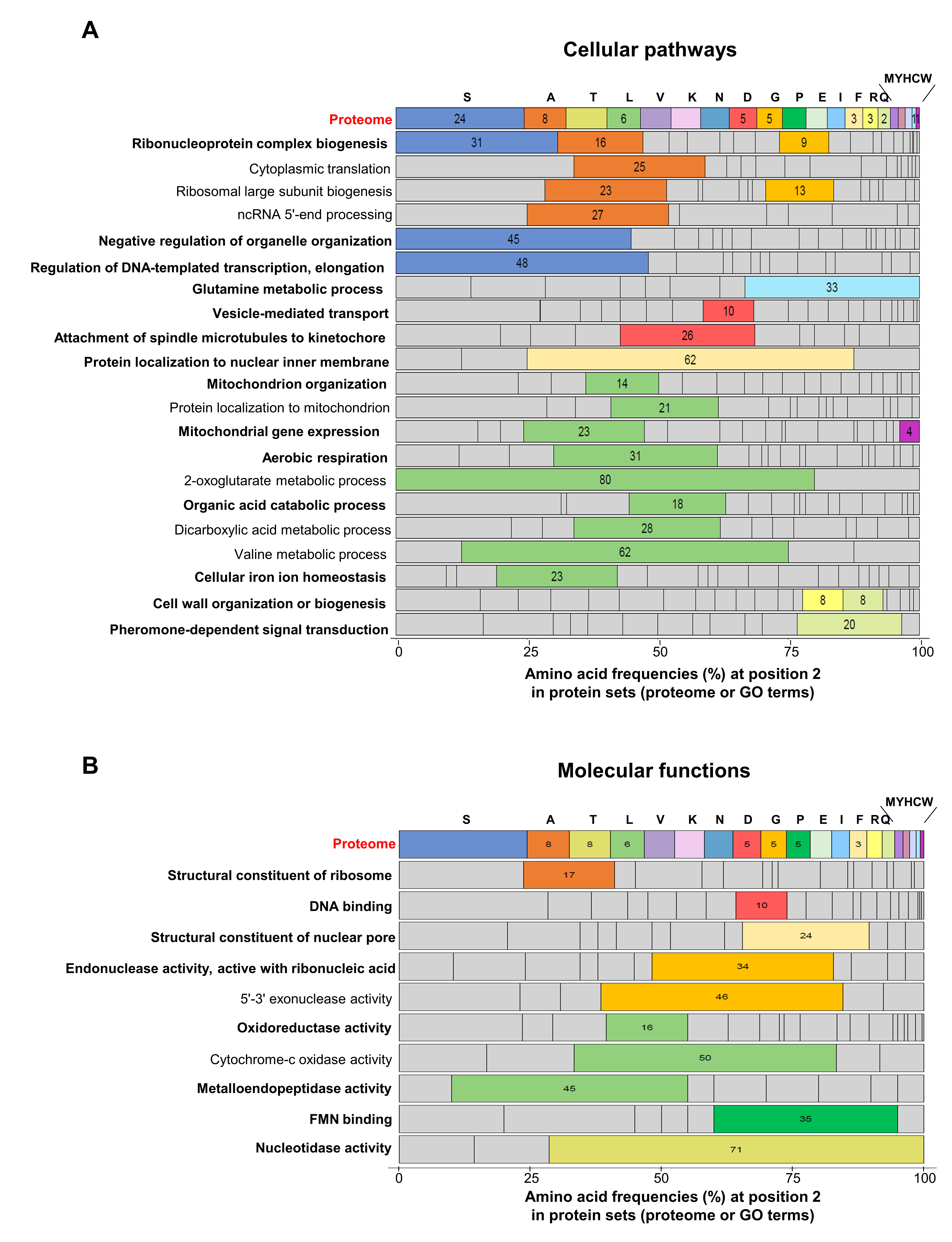

Supplement: S3 Fig — Bar graph showing the percentages of use of the 20 amino acids at position 2 in GO terms corresponding to cellular pathways (A) and molecular functions (B) showing preferences at position 2 for specific amino acids. All BestN (bold type) and BestF (normal type) GO terms selected by our algorithm are shown. Significant overrepresentations of amino acids (HGT score > 3) are highlighted. Amino acids are sorted in decreasing order of use at position 2 in the proteome. (TIF) [file pgen.1010848.s009.tif]

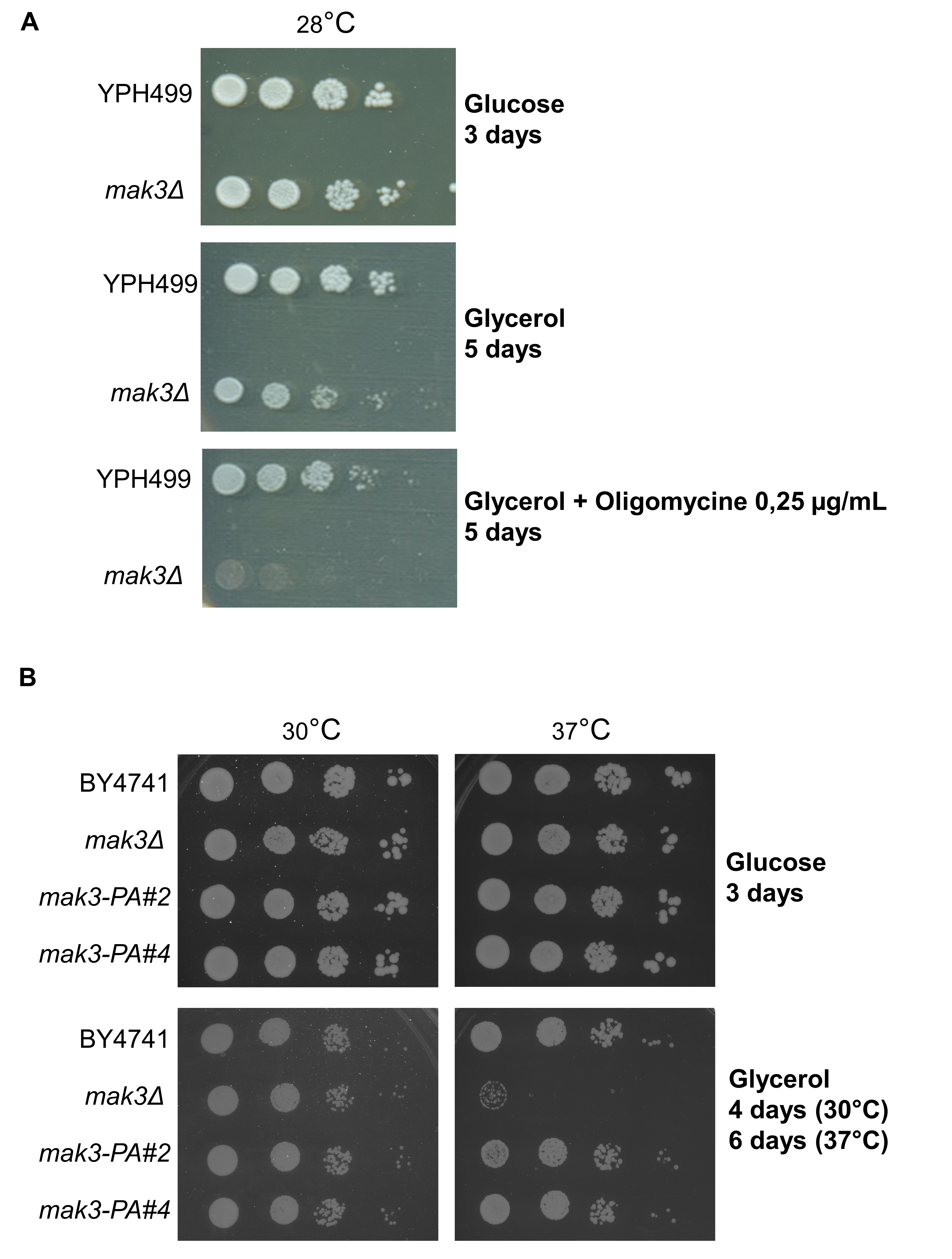

Supplement: S4 Fig — (A) The growth defect of the mak3Δ strain on glycerol medium is significantly enhanced by the inhibition of mitochondrial ATP synthase by oligomycin. (B) C-terminal PA Tag required for the sel-TRAP experiment did not disrupt cell growth on glycerol medium. Strains were grown on Glucose medium and diluted in water to 0.5 (A) or 0.2 (B) OD600. Tenfold serial dilutions were plotted (5μl) on glucose or glycerol medium optionally supplemented with oligomycin (0.25μg/μl). Temperature and incubation time are indicated. The mak3Δ and mak3-PA strains were derived respectively from the parental strains YPH499 and BY4741 (see Table A in S1 Supplementary Methods). (TIF) [file pgen.1010848.s010.tif]

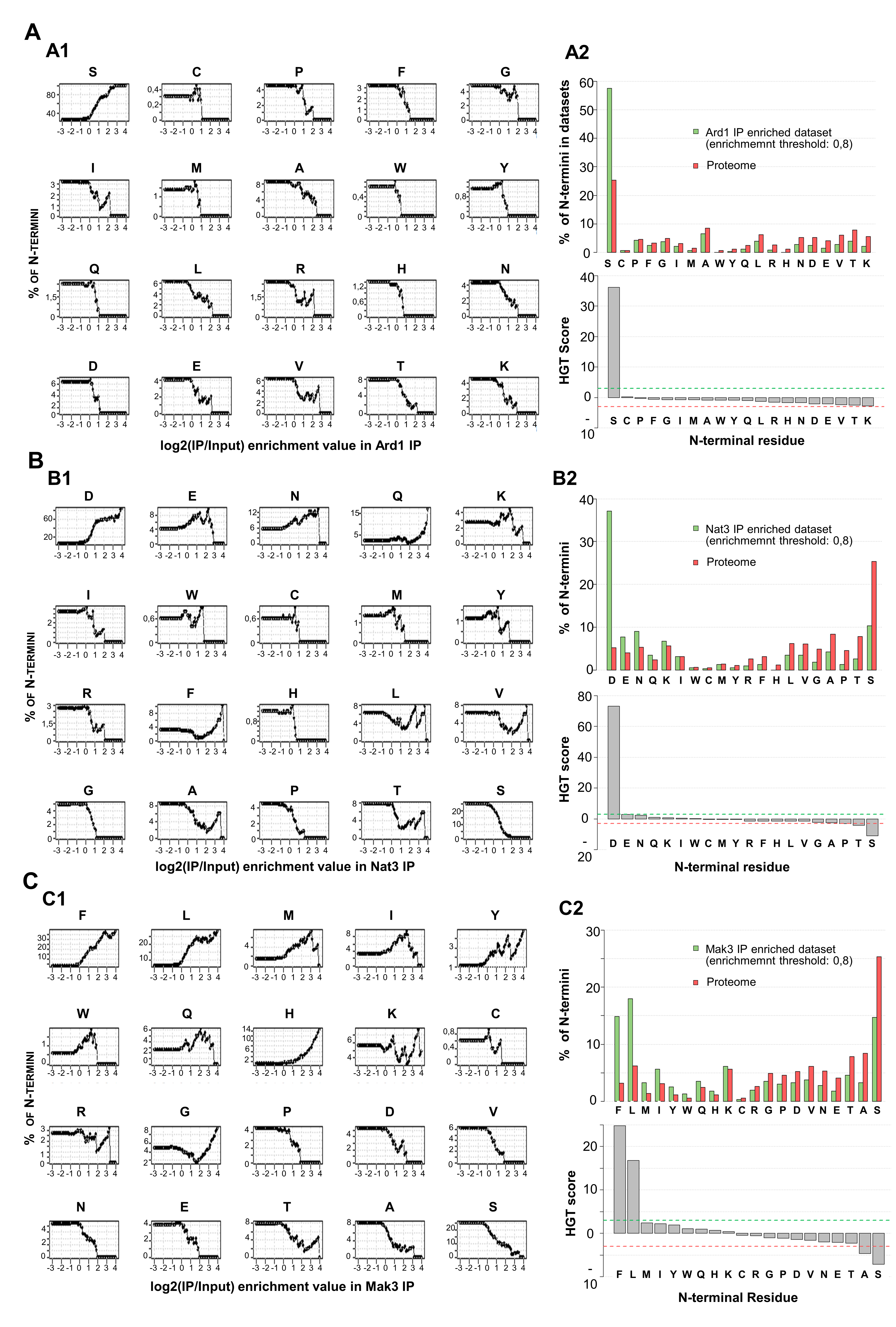

Supplement: S5 Fig — Analysis of the proportion of N-termini, defined as the amino acid observed at position 2, in proteins encoded in the transcriptomic data from Ard1-PA (A), Nat3-PA (B) and Mak3-PA (C) immunoprecipitation confirmed that sel-TRAP experiments have specifically purified canonical substrates of NatA, NatB and NatC respectively. Threshold-independent analyses showing for each amino acid the proportion of corresponding N-termini as a function of enrichment value in the sel-TRAP data are shown in A1, B1, and C1. For each x-axis value, the y-value shown is the % of N-termini among those encoded by mRNAs with an enrichment value greater than or equal to the x value. An enrichment value threshold of 0.8 was used to analyze a set of putative targets of each Nat (A2, B2, C2). The distribution of the different N-termini among putative targets (upper panel in A2, B2 and C2) showed enrichments for residues defining canonical substrates of each Nats (i.e. S at position 2 for NatA, D, N, E and Q at position 2 for NatB, and F, L, I and W at position 2 for NatC). Statistical evaluation of the observed enrichment was performed by calculating HGT scores (lower panel in A2, B2 and C2) and confirmed a significant enrichment at position 2 (HGT≥3, dotted line) in serine (S) for NatA, aspartate (D) for NatB, and phenylalanine (F) and leucine (L) for NatC. (TIF) [file pgen.1010848.s011.tif]

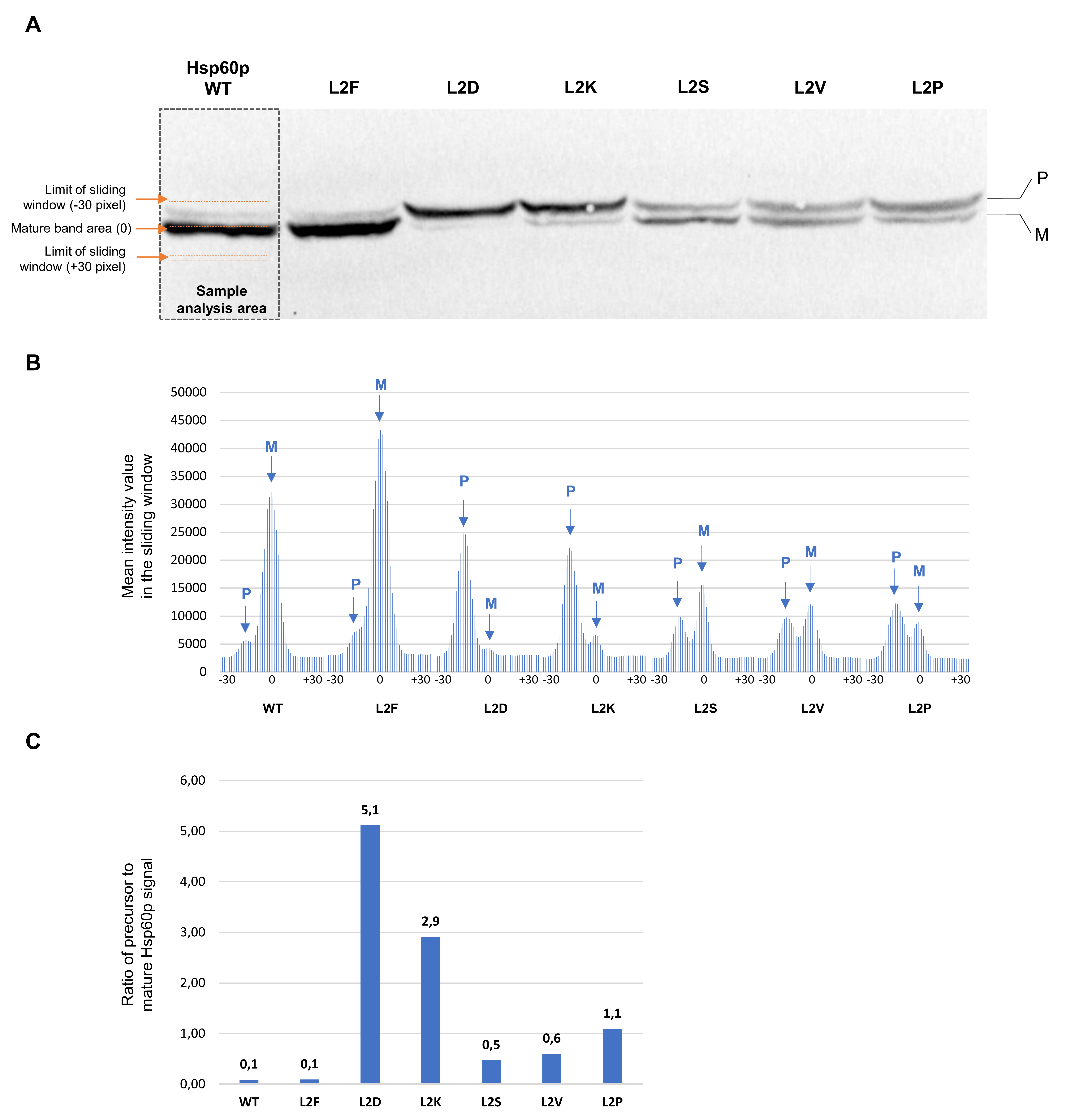

Supplement: S6 Fig — (A) The quantification procedure is illustrated on the western blot shown in Fig 5A1. We developed a basic ImageJ macro to collect in each sample area the average intensity values measured in a sliding window from 30 pixels upstream to 30 pixels downstream of the band corresponding to mature Hsp60p. (B) For each sample, the distribution of the average intensity value was plotted along the x-axis, allowing determination of the peak intensity for the precursor (P) and mature (M) bands. Because the precursor and mature intensity Gaussians overlap, we systematically corrected the precursor intensity value by the symmetric value on the x-axis to avoid overestimating the precursor band accumulation. (C) This accumulation was estimated by comparing the ratio of precursor to mature Hsp60p signal in the mutant strains compared to the reference WT strain. (TIF) [file pgen.1010848.s012.tif]

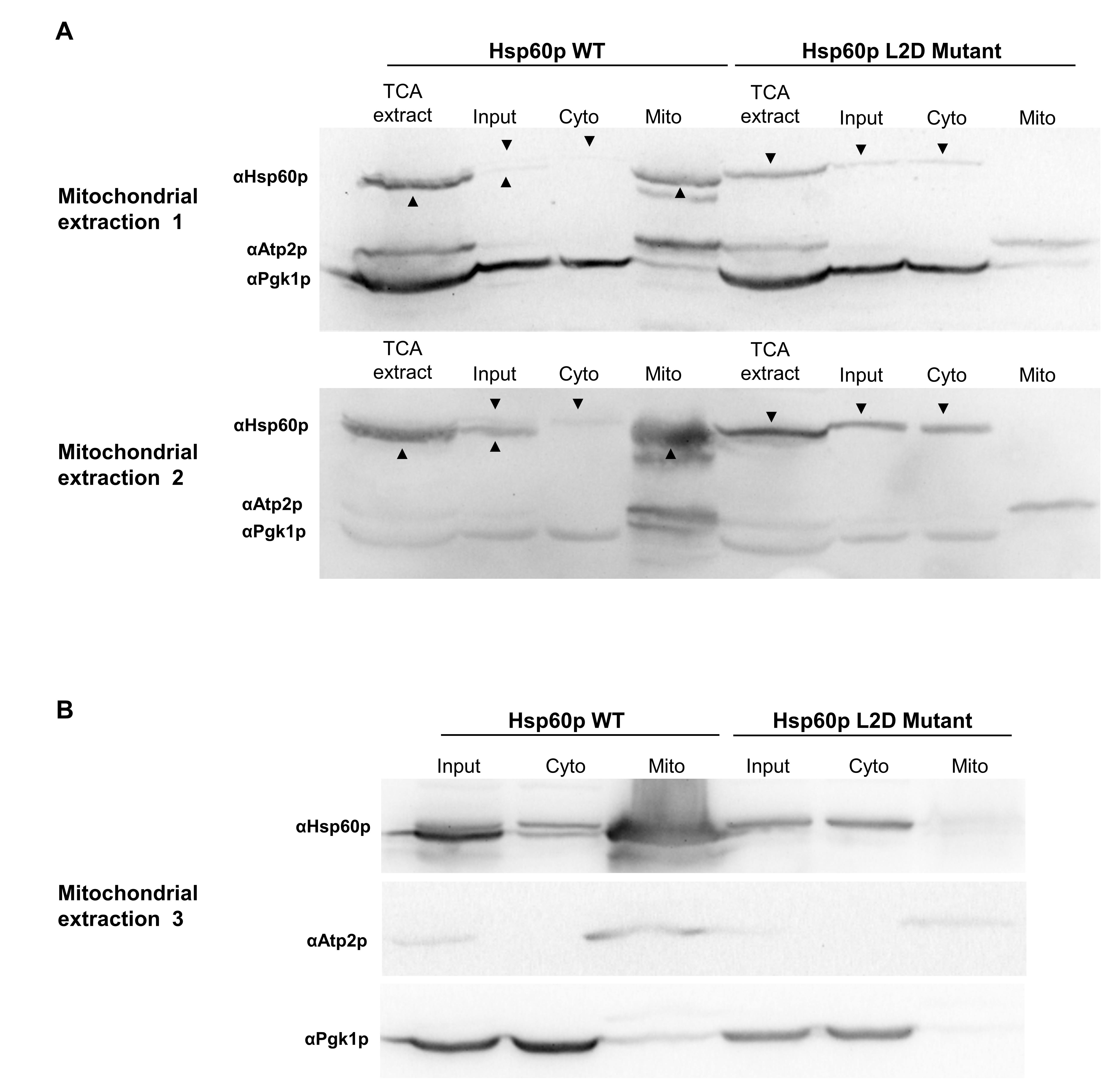

Supplement: S7 Fig — Cellular fractionations from three independent cultures were performed from the L2D mutant strain and its parental YPH499 pam16Δ-MAGN76D strain. The L2D mutant was chosen for these experiments because it presented the highest rate of accumulation of the precursor (closed to 80% of the total Hsp60 signal according Fig 5A2). Input protein samples were obtained by directly mixing spheroplasts after cell wall digestion with 2X Laemmli buffer. For comparison, crude TCA extracts were also obtained from the same culture before digestion. Cytosolic and mitochondrial protein samples were obtained after differential centrifugations by mixing the corresponding fractions with 2X Laemmli buffer. (A) Western blot images obtained after hybridization of antibodies directed against Hsp160p, Atp2p, and Pgk1p and simultaneous acquisition of the signals from the different antibodies. The downward and upward pointing arrows indicate, in each lane, the precursor and mature bands of Hsp60p respectively. Note that the TCA extraction method caused a shift in protein migration compared with the fractionation samples. Mature Hsp60p observed in WT strain is highly accumulated in mitochondrial fractions, whereas Pgk1p protein is mainly detected in the cytosolic fraction in both strain samples. Hsp60p precursor that highly accumulated in L2D mutant is only detected in the cytosolic fraction, confirming that it is not imported into mitochondria. (B) Western blot images obtained after independent antibody hybridizations. Before incubation with antibodies, the membrane was cut into three parts to separate the areas corresponding to the localization of Hsp160p, Atp2p and Pgk1p. This allowed us to improve the signal for each protein by optimizing the image acquisition time and to confirm the localization of the Hsp60p precursor in the cytosol. (TIF) [file pgen.1010848.s013.tif]

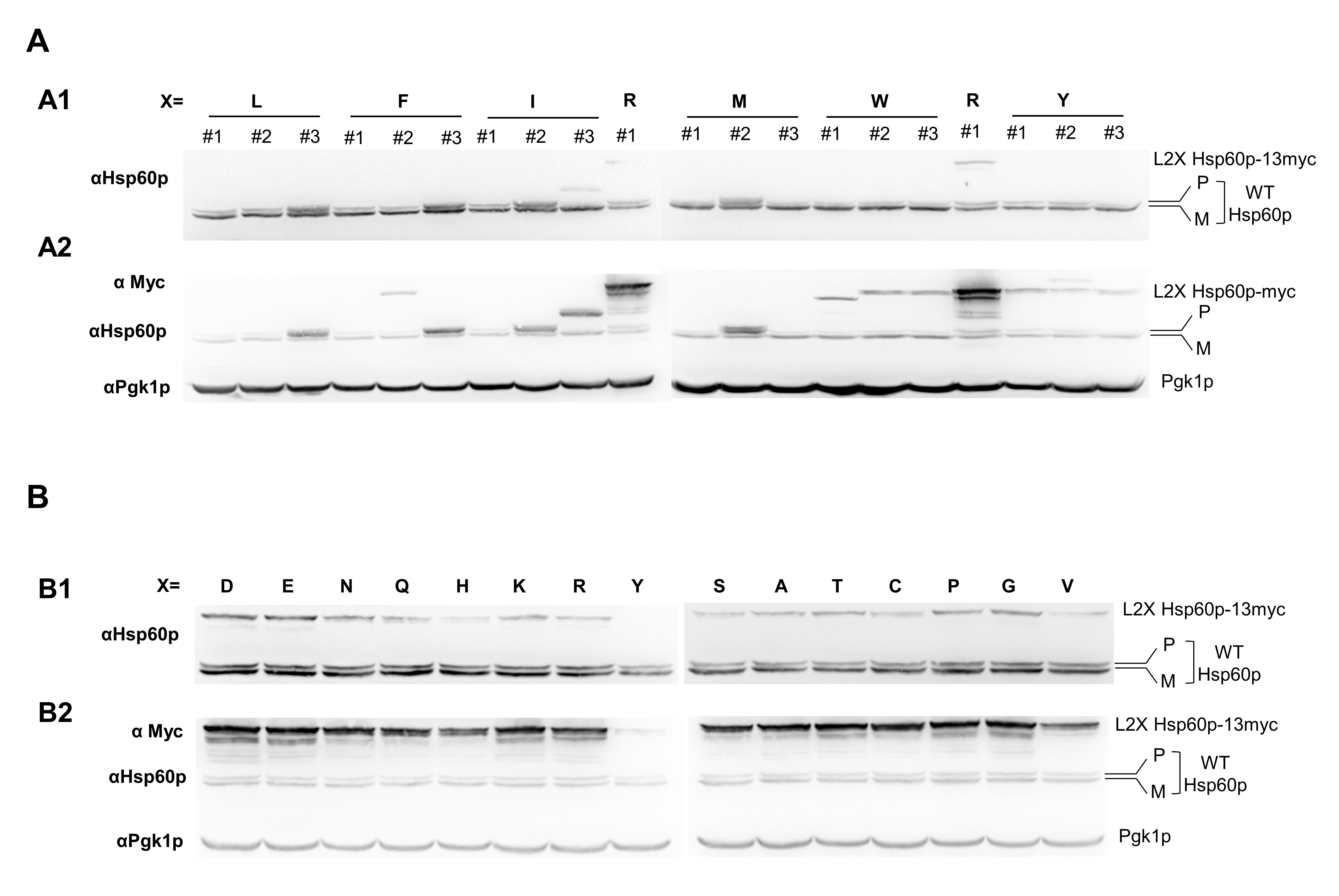

Supplement: S8 Fig — Protein extracts were analyzed by SDS-PAGE followed by western blots using antibodies against myc epitope, Hsp60p, and Pgk1p. The position of the precursor and mature Hsp60p are indicated. (A) Analysis of clones transformed with plasmid expressing a toxic Hsp60p-13myc protein (X = L, F, I, M, W, see Fig 5B) and of those obtained with the pHSP60(Y) for which a longer exposure time was necessary to detect the Hsp60p-13myc protein (see S8B Fig). (B) Analysis of clones transformed with plasmids that allow the rescue of the dominant negative toxicity of the Hsp60p-13myc protein (X = D, E, N, Q, H, K, R, Y, S, A, T, C, P, G, V, see Fig 5B). (TIF) [file pgen.1010848.s014.tif]
